# Exploring the level of metabolic reprogramming and the role of prognostic factor SF3A3 in hepatocellular carcinoma through integrated single-cell landscape analysis

**DOI:** 10.1371/journal.pone.0323559

**Published:** 2025-05-27

**Authors:** Wanshuo Wei, Yuan Gan, Xindan Zhang, Yumo Chen, Zengfeng Huang, Shuhan Wang, Xiaomei Xie, Yongle Li, Pengtao Qin, Lihe Jiang

**Affiliations:** 1 Scool of Clinical Medicine, School of Basic Medical Sciences, Youjiang Medical University for Nationalities, Baise, Guangxi Province, P.R. China; 2 The First Affiliated Hospital, School of Basic Medical Sciences, Guangxi Medical University, Nanning, Guangxi Province, P.R. China; 3 Fujian Key Laboratory of Medical Bioinformatics, School of Medical Technology and Engineering, Fujian Medical University, Fuzhou, Fujian Province, P.R. China; 4 Key Laboratory of Biomarkers and in Vitro Diagnosis Translation of Zhejiang province, Hangzhou, Zhejiang province, P.R. China.; Rutgers: Rutgers The State University of New Jersey, UNITED STATES OF AMERICA

## Abstract

This study aims to investigate metabolic reprogramming heterogeneity in hepatocellular carcinoma (HCC) cells and identify novel therapeutic targets for HCC treatment. Single-cell RNA sequencing data from public databases were used to analyze the TME of HCC and reveal the characteristics of different cell subsets, including mononuclear phagocytes, epithelial cells, endothelial cells, NK/T cells, B cells, and unknown cells. The analysis revealed that these cell subsets play their own unique roles in tumor progression and immune escape. Analysis of copy number variations (CNVs) was performed on tumor-derived epithelial cells, with the epithelial cells in Cluster 3 subgroup showing the highest CNV levels. Gene Ontology (GO) enrichment analysis revealed that these cell subsets were involved in a variety of biological processes such as immune response, cell communication, and metabolic pathways, which were consistent with their functional roles. Pseudotemporal analysis further delineated the malignant trajectory of HCC cells, with Cluster 3 exhibiting enhanced phosphatidylinositol metabolism, suggesting a critical role for metabolic reprogramming in tumor invasion and proliferation. Furthermore, a diagnostic model incorporating metabolic reprogramming-associated gene signatures was established, which effectively distinguished HCC from normal tissues. Among these signatures, splicing factor 3a subunit 3 (SF3A3) was identified as both diagnostic and independent prognostic biomarker. Mechanistically, SF3A3 knockdown in HCC cell lines significantly suppressed proliferation, migration, PI3K/AKT signaling, and EMT marker expression, thereby demonstrating its role in driving HCC aggressiveness. In conclusion, these findings elucidate novel molecular characteristics of HCC based on metabolic reprogramming, while establishing SF3A3 as a promising multi-faceted target for HCC diagnosis, prognostic assessment, and therapeutic intervention.

## Introduction

Hepatocellular carcinoma (HCC) is one of the malignancies with a high incidence and mortality rate worldwide [1]. According to global cancer statistics, HCC ranks first in incidence among liver tumors, and its associated mortality has been steadily increasing over the years [2]. The development of HCC is related to various risk factors, including chronic hepatitis virus infection, cirrhosis, alcohol abuse, and metabolic syndrome [3].

Metabolic reprogramming refers to extensive adjustments made by cells to their energy and material metabolism under specific physiological or pathological conditions [4]. In recent years, metabolic reprogramming has been increasingly recognized for its significance in the initiation and progression of HCC [5]. Metabolic reprogramming leads to the biological heterogeneity of HCC, causing it to exhibit distinct biological characteristics and clinical outcomes in different patients [6]. In HCC, metabolic reprogramming is primarily manifested as significant alterations in glucose, lipid, and amino acid metabolic pathways [7,8]. These metabolic changes enable tumor cells to favor aerobic glycolysis, even under normoxic conditions [9].

Phosphatidylinositol and its phosphorylated derivatives serve as pivotal signaling molecules that orchestrate diverse cellular processes via dynamic metabolic remodeling [10]. Aberrant phosphatidylinositol remodeling promotes tumor progression through constitutive activation of oncogenic pathways, particularly the PI3K/AKT/mTOR axis, and extensive metabolic reprogramming [11,12]. The PI3K/AKT pathway plays a crucial role in metabolic reprogramming in HCC [13]. This signaling axis promotes the Warburg effect and metabolic reprogramming in HCC through coordinated activation of glycolytic enzymes HK2, PFK-1 and PKM2, concurrent suppression of PDK1-dependent mitochondrial oxidative metabolism, and synergistic integration with oncogenic signals including CD36/Src upregulation, SHH overexpression and PTEN loss [14]. These oncogenic metabolic reprogramming are mediated by the precisely regulated phosphorylation of phosphatidylinositol by specific phosphatidylinositol kinases, with phosphoinositide 3-kinase (PI3K) serving as the central effector that coordinates metabolic alterations with proliferative signaling cascades in HCC [15].

The rapid advancement of single-cell RNA sequencing (scRNA-seq) technology has paved the way for systematic dissection of distinct metabolic landscapes in diverse cell populations within HCC [16]. In our study, through integrating single-cell multi-omics data to systematically identify critical metabolic reprogramming-associated gene signatures and verify SF3A3’s function in HCC cells, we found that obvious metabolic heterogeneity exists within tumor epithelial cells, and SF3A3 can serve as a biomarker for diagnosis and prognosis assessment as well as a potential therapeutic target in HCC.

## Materials and methods

### Tumor data acquisition

The single-cell sequencing data and gene expression data used in this study were obtained from the Gene Expression Omnibus (GEO), The Cancer Genome Atlas (TCGA), and the International Cancer Genome Consortium (ICGC). From the GEO database, we downloaded the following gene expression datasets: GSE22058 (100 tumor samples, 97 normal samples), GSE112790 (183 tumor samples, 15 normal samples), GSE101685 (24 tumor samples, 8 normal samples), GSE84402 (14 tumor samples, 14 normal samples), GSE62232 (81 tumor samples, 10 normal samples), GSE121248 (70 tumor samples, 37 normal samples), GSE36376 (240 tumor samples, 193 normal samples), GSE14520 (225 tumor samples, 220 normal samples), GSE76427 (115 tumor samples, 52 normal samples), and GSE10143 (80 tumor samples, 307 normal samples). Single-cell datasets GSE149614 and GSE151530 were also retrieved from the GEO database. Gene expression profiles and clinical information of 369 HCC samples and 50 adjacent normal tissue samples were obtained from the TCGA database, and expression profiles and clinical information for 243 tumor tissues and 202 normal tissues were downloaded from the ICGC database.

### Preliminary scRNA-seq analysis

Single-cell RNA sequencing (scRNA-seq) analysis was conducted using datasets GSE149614 and GSE151530. During the processing of the scRNA-seq data, we retained high-quality cells that meet the following criteria: mitochondrial gene expression accounted for less than 10%, red blood cell gene expression was under 3%, and the number of expressed genes ranged from 200 to 8000. We used the three-step “NFS” strategy (NormalizeData, FindVariableFeatures, and ScaleData) for data normalization. Cell cycle gene normalization was performed using the “cc.genes.updated.2019” dataset from the “Seurat” package, and potential doublets were removed using the “DoubletFinder” package. The remaining cells were processed to detect 2000 highly variable genes using the “FindVariableFeatures” function. Subsequently, principal component analysis (PCA) was used to reduce the dimensionality of the scRNA-seq data. To eliminate batch effects between samples, the “Harmony” package was employed to perform soft k-means clustering. The single-cell data were split by origin into tumor and normal cells, which were then clustered separately. Cell clustering was carried out using the “FindClusters” function with a resolution parameter set to 0.31. Ultimately, 28,906 cells were classified as tumor cells, and 7,694 cells were categorized as normal cells. Cells were annotated and divided into various subgroups based on the results of dimensionality reduction and clustering. Epithelial cell subgroups were extracted, and epithelial subgroups derived from tumor cells and normal cells were included in the “inferCNV” package for computation. To accurately identify malignant cells that exhibit clonally extensive chromosomal copy number variations (CNV), the epithelial subgroups derived from normal cells were used as references to infer CNV profiles. K-means clustering was applied to the inferCNV results for secondary clustering, and the final number of clusters was determined using both the Elbow Method and Betweenness Analysis to ensure optimal clustering performance. CNV scores for each cluster were calculated and visualized.

### Enrichment analysis and cell trajectory analysis

To explore the functional and pathway enrichment of each subgroup, we extracted the top 200 genes with the highest expression levels in each subgroup. Gene Ontology (GO) analysis and Kyoto Encyclopedia of Genes and Genomes (KEGG) pathway enrichment analysis were performed using the “clusterProfiler” package in R, with a p-value threshold set to 0.05. Cell developmental trajectories of the subgroups were inferred using the Monocle2 package. After dimensionality reduction and cell sequencing, cell differentiation paths were derived based on standard parameters. The algorithm is based on a simple yet effective principle: cells are clustered first, and trajectories are constructed according to the average central point of the cell population. At the same time, the distance of other cells to the presumed trajectory is calculated, and these cells are assigned to the nearest cell group.

### Metabolic level analysis

We used the “scMetabolism” package to identify and analyze metabolic pathways in single cells, providing an in-depth investigation of the metabolic levels between subgroups [17]. The “scMetabolism” R package utilizes metabolic pathway databases including KEGG and Reactome to quantify pathway activity through computational algorithms such as ssgsea and AUCell.

### Metabolic gene signature selection

To identify the most malignant metabolic gene signatures, guiding research on metabolic reprogramming in HCC, we selected the single-cell subgroups with the highest CNV scores after performing inferCNV and K-means clustering, and extracted marker genes for further study. We obtained metabolic gene sets from KEGG and Reactome databases within the “scMetabolism” package and intersected the marker genes with these gene sets for further analysis [17,18].

Within the sample, the order of gene expression can follow two patterns: G_i_ > G_j_ or G_i_ < G_j_. Here, G_i_ > G_j_ indicates that the expression level of gene i is higher than gene j, while G_i_ < G_j_ means that the expression level of gene i is lower than gene j. For specific types of tissue samples, we first rank the gene expression abundance within the sample, and then calculate the proportion P(G_i_ > G_j_) for each gene pair (e.g., gene i and gene j) in the sample. The probabilities P(G_i_ > G_j_) and P(G_i_ < G_j_) for gene expression comparisons are defined by Equations (1) and (2):


$$P\left(\text{X}>\text{Y}\right)=\frac{1}{n}\sum_{\text{z}=1}^{n}I\left[X_{\text{z}}>Y_{\text{z}}\right]$$ (1)

$$P\left(\text{X}<\text{Y}\right)=\frac{1}{n}\sum_{\text{z}=1}^{n}I\left[X_{\text{z}}<Y_{\text{z}}\right]$$ (2)

In this context, “n” denotes the total number of normal samples or tumor samples, and “I” represents the indicator function. We define gene pairs that satisfy the conditions P(G_i_ > G_j_) or P(G_i_ < G_j_) greater than 0.9 in a class of samples (normal or tumor) as highly stable gene pairs for that class of samples. In the training set, if a gene pair maintains the REO (Relative Expression Order) pattern (i.e., G_i_ > G_j_ or G_i_ < G_j_) in more than 90% of tumor samples, while showing the opposite pattern in more than 90% of normal samples, the gene pair is termed a stable reversal gene pair between the two types of samples [19]. Subsequently, based on the gene expression profiles and stable reversal gene pairs, we represent G_i_ > G_j_ as 1 and G_i_ < G_j_ as 0, thus generating a new expression profile. The datasets GSE22058, GSE112790, GSE101685, GSE84402, GSE62232, GSE121248, and GSE36376 were used as training group 1. The intersection between training group 1 and previously extracted metabolic genes was calculated to identify the stable reversal gene pairs. The stable reversal gene pairs and the expression matrix of training group 1 were extracted, generating a new expression matrix for further analysis.

### Diagnostic model

The data from five cohorts, GSE14520, GSE76427, GSE10143, TCGA, and ICGC, were used as validation group 1. Expression of the stable reversal gene pairs were evaluated in validation group 1. Both training group 1 and validation group 1 were incorporated into a set of 117 machine learning models, which included nine different machine learning methods: Enet, GBM, SVM, RF, glm, plsRglm, Ridge, Lasso, and rpart. The models were ranked based on the average AUC values from both the training and validation groups [20].

### Prognostic performance analysis

Genes involved in the stable reversal gene pairs were assessed for prognostic capability in the TCGA and ICGC cohorts, with TCGA serving as training group 2 and ICGC as validation group 2. A univariate and multivariate COX analysis was performed on the constituent genes of stable reversal gene pairs, and Kaplan-Meier survival curves were used to evaluate patient risk groups.

### Cell line expression validation and immunohistochemistry

The HCC cell line MHCC97-H and normal human liver cell line THLE-2 were purchased from Cell Bank of Type Culture, Shanghai Institute of Biochemistry and Cell Biology, Chinese Academy of Sciences. To assess SF3A3 expression, quantitative real-time PCR (qRT-PCR) experiments were conducted in HCC cell line MHCC97-H and normal human liver cell line THLE-2. For cell culture, DMEM medium supplemented with 10% fetal bovine serum and 1% penicillin-streptomycin double antibiotics was used, and the cells were cultured in a humidified incubator at 37°C with 5% CO_2_. Knockdown of SF3A3 was performed in the MHCC97-H cell line using siRNA transfection with Lipofectamine 3000 (Thermo Fisher Scientific). Cells were seeded in six-well plates at a density of 2 × 10⁵ cells per well and incubated with the lipid suspension for 24 hours, followed by subsequent phenotypic assays. For the gene SF3A3, an siRNA was designed with the sense strand sequence of 5’-GGACCAGAUCAAUUCUGAUTT-3’ and the antisense strand sequence of 5’-AUCAGAAUUGAUCUGGUCCTT-3’. Immunohistochemical (IHC) slices of the target gene in liver cancer tissues and normal liver tissues were obtained from The Human Protein Atlas (HPA) to assess the expression of SF3A3.

### qRT-PCR

Total RNA was extracted from the cell lines using the RNA isolation kit (Axygen, Suzhou, China). The total RNA was then reverse transcribed into cDNA. qRT-PCR was performed using the SYBR Green reagent kit, and amplification was carried out on a thermal cycler (Bio-Rad, Hercules, CA, USA) with the following conditions: 95°C for 600 seconds, 95°C for 10 seconds, 65°C for 60 seconds, 97°C for 1 second, and 37°C for 30 seconds, for a total of 40 cycles. GAPDH was used as the internal reference, and the expression level of SF3A3 was quantified using the 2^−ΔΔCt^ method. To detect the expression level of SF3A3 using qRT-PCR, a primer pair was designed with the forward primer having the sequence 5’-CAAGCAAGCCAGGACAGGAGAAG-3’ and the reverse primer having the sequence 5’-AATAGGTTTGCCATCCCAGCCAAG-3’.

### Cell proliferation assay

Transfected cells were seeded into 96-well plates at a density of 2 × 10³ cells per well. After incubating for 24, 48, 72, and 96 hours, cell proliferation was assessed by adding Cell Counting Kit-8 (CCK-8) reagent and incubating at room temperature for 2 hours. Absorbance at 450 nm was measured with a microplate reader. The cell proliferation curve was fitted based on absorbance changes, and the proliferation inhibition rate was calculated post-transfection.

### Scratch wound healing assay

Transfected cells were seeded into six-well plates at a density of 3 × 10⁵ cells per well and incubated for 24 hours until cells adhered and reached full confluence in the plate. A 10 µL pipette tip was used to create a vertical scratch in each well, and cell debris was washed off with PBS. Images were captured under an inverted microscope at 0 and 24 hours to assess scratch closure.

### Colony formation assay

Transfected cells were seeded in six-well plates at a density of 5 × 10^2^ cells per well. After adding culture medium, the cells were incubated undisturbed for 10 days, with media changes every three days. At the end of the incubation, cells were fixed with 4% paraformaldehyde (PFA) and stained with 0.1% crystal violet solution.

### Western blot

After the cells were grown to the appropriate density, the culture medium was aspirated from six-well plates, followed by repeated gentle washing with phosphate-buffered saline (PBS) to ensure complete removal of residual medium. Adherent cells were mechanically detached using sterile scrapers, and the cell suspension was transferred to a centrifuge tube. The centrifuge tube was placed on ice and the cells were fully lysed by adding an appropriate amount of RIPA lysate containing protease inhibitors and phosphatase inhibitors. The mixture was incubated on ice for 30 min, during which time it was gently reversed and mixed at 10-min intervals. The samples were then centrifuged at 12000 rpm for 15 min at 4°C, and the supernatant was used as the total protein extract. The protein concentration was calculated by BCA method to ensure that the actual amount of protein added was consistent between different samples. Proteins were separated by SDS-PAGE, and the electrophoresed proteins were transferred from the gel to a PVDF membrane. Membranes were blocked with TBST buffer containing 5% bovine serum albumin (BSA) for 1h at room temperature. After incubating the primary and secondary antibodies, the protein bands were observed by chemiluminescence reagent (ECL), and the expression levels of target proteins were analyzed by image analysis system. The following antibodies were used in this article: SF3A3 (SF3A3 Polyclonal antibody, 1:3000, Proteintech, Cat No: 12070–1-AP), N-cadherin (N-cadherin Polyclonal antibody, 1:5000, Proteintech, Cat No: 22018–1-AP), E-cadherin (E-cadherin Polyclonal antibody, 1:5000, Proteintech, Cat No: 20874–1-AP), Vimentin (Vimentin Polyclonal antibody, 1:20000, Proteintech, Cat No: 10366–1-AP), GAPDH (GAPDH Monoclonal antibody, 1:20000, Proteintech, Cat No:60004–1-Ig), PI3K (PI3 Kinase p85 Beta Recombinant antibody, 1:500, Proteintech, Cat No: 83606–5-RR), p-PI3K (Phospho-PI3-kinase p85- alpha/ gamma (Tyr467/199) pAb, 1:1000, Abmart, Cat No: T40116S), AKT (AKT Polyclonal antibody, 1:5000, Proteintech, Cat No: 10176–2-AP), p-AKT (Phospho-AKT (Ser473) Monoclonal antibody, 1:5000, Proteintech, Cat No: 66444–1-Ig), Rabbit secondary antibody (HRP-conjugated Goat Anti-Rabbit IgG(H + L), 1:7500, Proteintech, Cat No: SA00001–2), Mouse secondary antibody (HRP-conjugated Goat Anti-Mouse IgG(H + L), 1:7500, Proteintech, Cat No: SA00001–1).

### Immunofluorescence

Immunofluorescence (IF) experiments achieve the localization and quantitative analysis of target proteins in cells by antigen-antibody specific binding and fluorescent labeling technology. The experimental materials included 4% paraformaldehyde fixative, 0.1% Triton X-100 permeabilizing agent, TBST, 5% bovine serum albumin blocking solution, specific primary antibody and fluorescence-labeled secondary antibody, DAPI nuclear staining and anti-fluorescence quenching plate sealant. After fixation with 4% paraformaldehyde, the cells were permeabilized with Triton X-100 for 10 min at room temperature and subsequently blocked with BSA for 30min. After completion of blocking, the primary antibody was incubated overnight, and the secondary antibody was incubated at room temperature in the dark for 1 hour. After completion of incubation, DAPI was used for nuclear staining for 10min. Primary antibody, secondary antibody and DAPI were washed three times with TBST for 5 min each at the end of incubation. Microscopic observations and photographs were taken within 1 h after addition of anti-fluorescence quench sealant.

### Statistical analysis

Bioinformatics analysis and related figure plotting were performed using R (v4.4.0). Statistical analysis of experimental data and figure creation were done using GraphPad Prism (v9.0). ImageJ was used to analyze the experimentally generated pictures. Comparisons between two groups were conducted using a t-test, with a p-value < 0.05 considered statistically significant. *P < 0.05, **P < 0.01, ***P < 0.001,****P < 0.0001.

## Results

### Cell subgroup distribution in hepatocellular carcinoma

**Fig 1:**
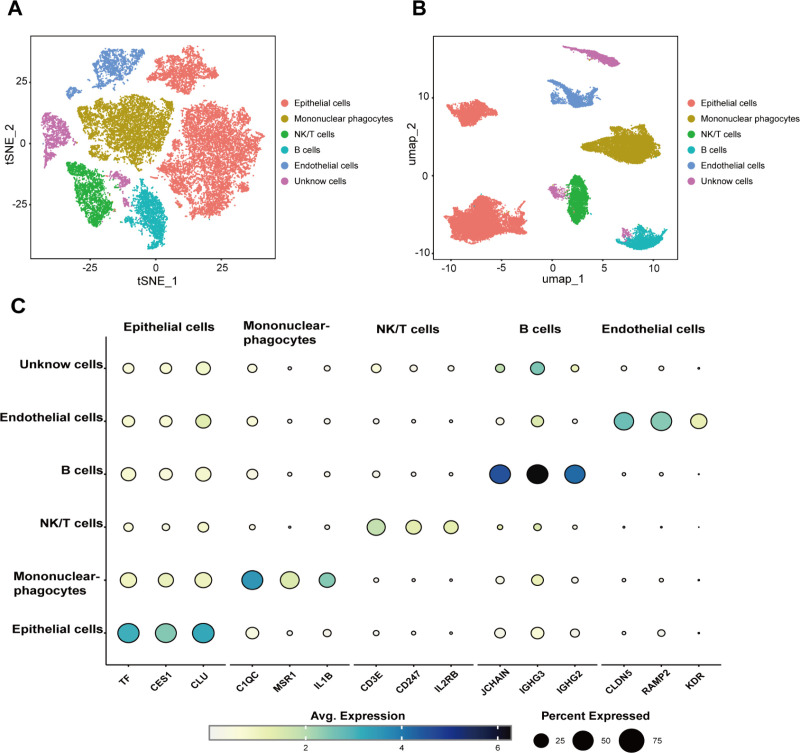
Distribution of single-cell subsets. **(A)** Single-cell t-SNE map. **(B)** Single-cell UMAP map. **(C)** Marker genes were annotated for each cell type.

**Fig 2:**
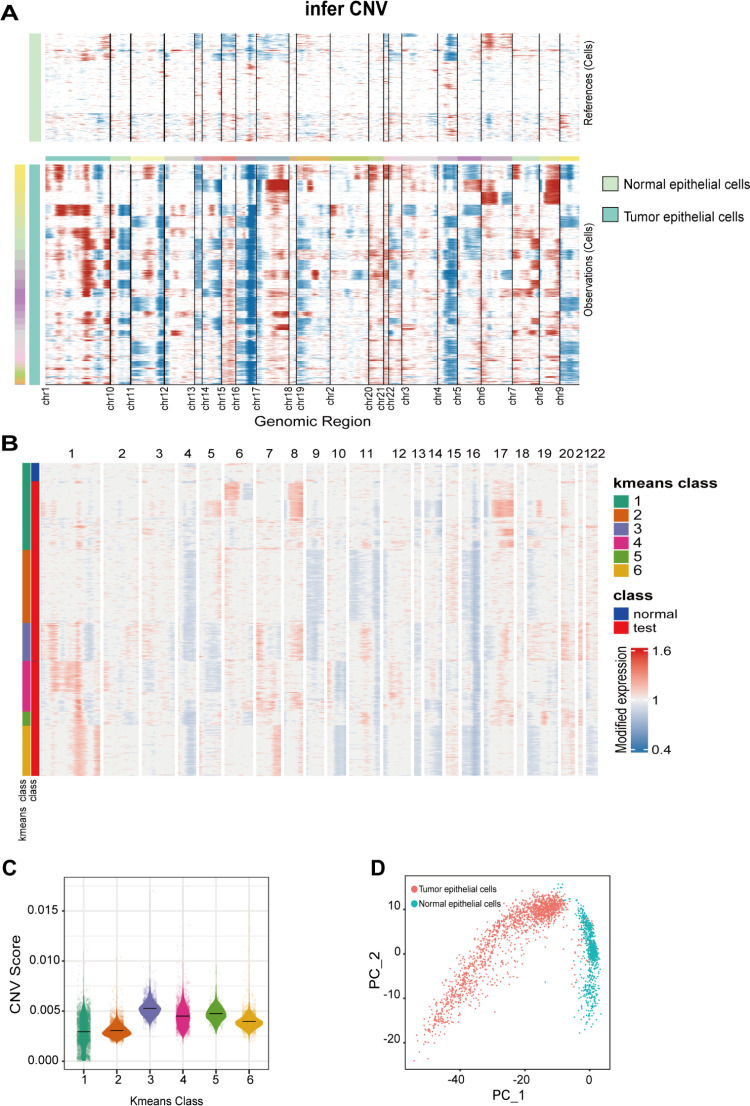
Inferring CNV clustering and scoring. **(A)** Results of CNV in epithelial derived tumor cells. **(B)** K-means diagram of the elbow. **(C)** CNV scores for each cluster. **(D)** PCA dimensionality reduction map of normal and tumor cells used for inferCNV.

To clarify the distribution of cells in HCC, we performed dimensionality reduction clustering on single-cell data and annotated the single-cell subgroups using marker genes (S1 Fig). The cell subgroups in HCC were primarily classified into mononuclear phagocytes, epithelial cells, endothelial cells, NK/T cells, B cells, and some unknown cells (Fig 1A-1C). It is worth noting that a subset of cellular populations remains unclassified because they cannot be well grouped by existing markers, which may be caused by statistical errors or poor marker resolution accuracy. We excluded the unknown cell subgroup and included epithelial cells from tumor tissue and normal tissue for analysis using the “inferCNV” program. The results revealed that the CNV levels of epithelial cells from tumor tissue were significantly higher than those from normal tissue (Fig 2A). The results of the CNV analysis were clustered using K-means, and the optimal number of clusters was determined through an elbow plot combined with betweenness analysis (S2 and S3 Figs) [21]. After being divided into six clusters, cells with similar CNV levels were grouped together, showing considerable differentiation between clusters (Fig 2B). To facilitate further analysis, the six clusters separated by unsupervised clustering were identified as 1, 2, 3, 4, 5, and 6, respectively. To better quantify the CNV variation levels between clusters, we calculated the CNV scores for each cluster and found that Cluster 3 exhibited the highest CNV variation (Fig 2C) [21–24]. The PCA dimensionality reduction map showed that tumor cells and normal cells did not gather too much together. Therefore, it can be considered that there are not too many tumor cells mixed with normal cells, which can be used as a reference group for inferCNV (Fig 2D).

### Functional enrichment analysis of cell subgroups

**Fig 3:**
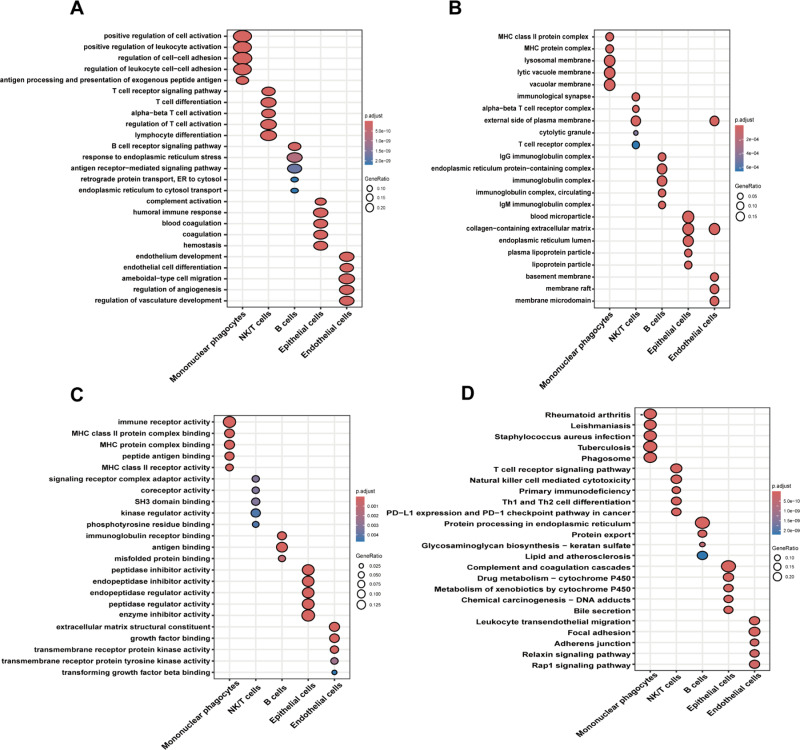
GO and KEGG enrichment analyses of single-cell subsets. **(A)** Biological process enrichment of individual cell subsets. **(B)** Cellular components enrichment of individual cell subsets. **(C)** Molecular functions enrichment of individual cell subsets. **(D)** KEGG pathway enrichment analysis for each cell subtype.

We performed GO functional enrichment analysis and KEGG pathway enrichment analysis on the various single-cell subgroups to explore the pathways enriched in different cell types in HCC, while also validating the accuracy of our single-cell subgroup definitions. We found that mononuclear phagocytes were mainly enriched in functions related to regulation of leukocyte cell-cell adhesion, MHC protein complex, and MHC protein complex binding. NK/T cells were primarily enriched in functions such as regulation of T cell activation, immunological synapse, and kinase regulator activity. B cells were mainly enriched in functions related to the B cell receptor signaling pathway, IgG immunoglobulin complex, and immunoglobulin receptor binding. Endothelial cells were primarily enriched in functions like endothelium development, basement membrane, and extracellular matrix structural constituent (Fig 3A-3C). KEGG pathway enrichment analysis was used to calculate the pathway enrichment of each subgroup of cells, and the results were analyzed combined with GO functional enrichment results. Our enrichment analysis results aligned with the expected functional characteristics of each cell type, confirming the robustness of our single-cell subpopulation classification (Fig 3D).

### Cell communication and metabolic reprogramming in hepatocellular carcinoma

**Fig 4:**
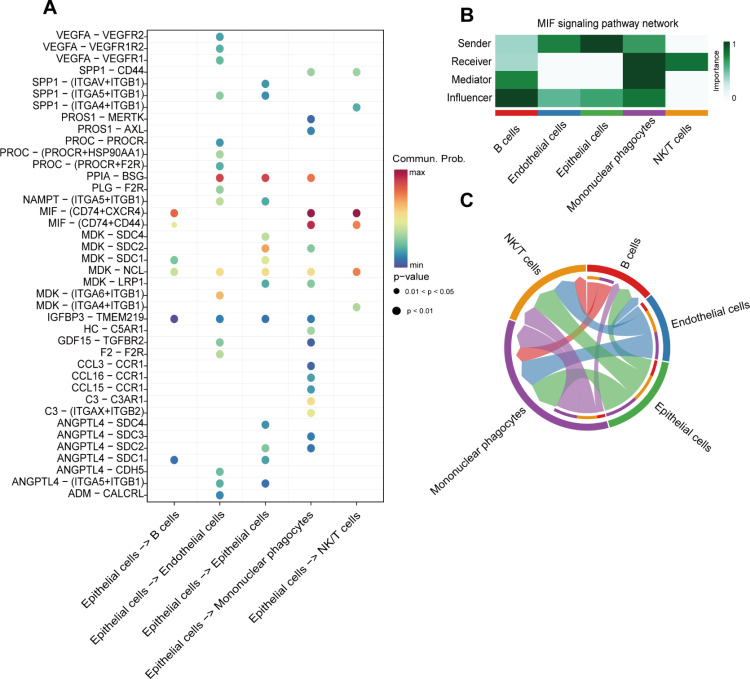
Cell communication. **(A)** Major signaling pathways for epithelial cell communication. **(B)** MIF signaling pathway communication network. **(C)** MIF signaling pathway communication of cell subsets.

The growth of tumor cells is closely related to tumor microenvironment. We aimed to explore cell communication in tumor epithelial cells to elucidate changes in the tumor microenvironment. We found that MIF (CD74-CXCR4) and MIF (CD74-CD44) were significantly upregulated in tumor epithelial cells. Tumor epithelial cells affect mononuclear phagocytes, NK/T cells, and B cells primarily through these two signaling pathways (Fig 4A). All cell-to-cell communication networks were established using weighted directed network analysis methods. Epithelial and endothelial cells mainly act as senders and influencers of the MIF signaling pathway, affecting immune cells (Fig 4B and 4C). This may be a key factor in immune escape of tumor cells.

**Fig 5:**
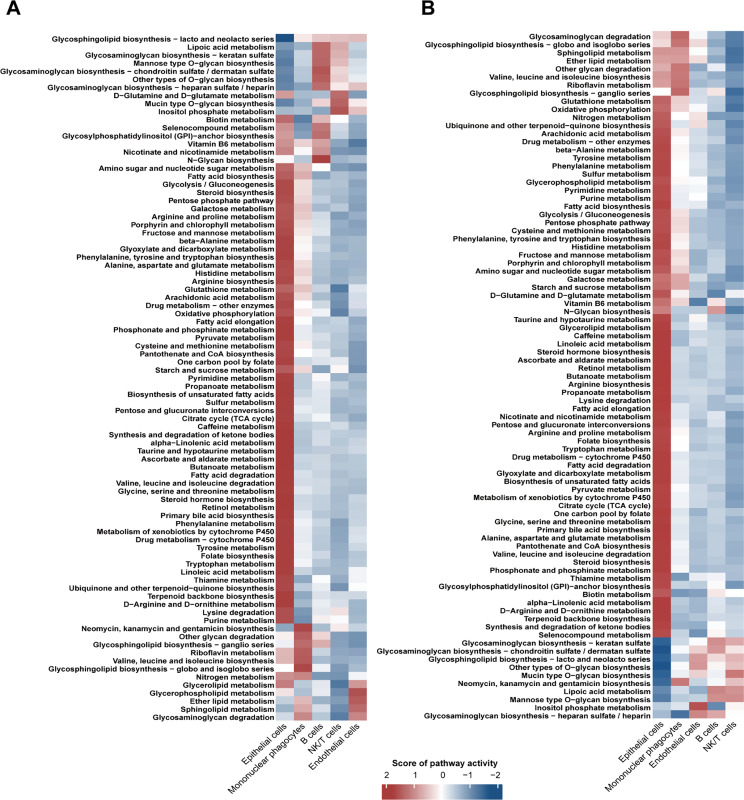
Metabolic analysis. **(A)** The AUCell method was used to calculate the level of cellular metabolism. **(B)** The ssgsea method was used to calculate the level of cellular metabolism.

To explore metabolic reprogramming in HCC, we performed metabolic assays. We found that epithelial cells showed significantly greater metabolic changes compared with other cell types in HCC (Fig 5A and 5B).

### Metabolic reprogramming changes and developmental trajectory of malignant cells

**Fig 6:**
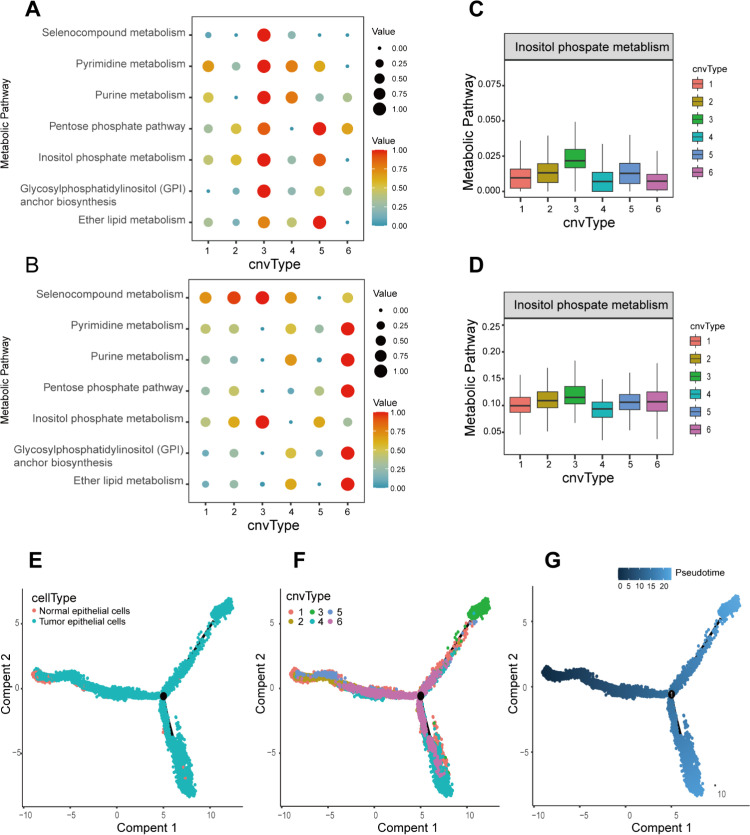
Metabolic levels and developmental trajectories of malignant cells. **(A)** Metabolic activity analysis of CNV subgroups using KEGG database and AUCell method. **(B)** Metabolic activity analysis of CNV subgroups using KEGG database and ssgsea method. **(C)** Inositol phosphate metabolism was calculated using Reactome database and AUCell method. **(D)** Inositol phosphate metabolism was calculated using Reactome database and ssgsea method. **(E)** Plot of developmental trajectories of tumor versus normal epithelial cells. **(F)** Plot of developmental trajectories stratified by CNV subgroups. **(G)** Temporal trajectories of cell development.

We performed metabolic level analysis on each cell subgroup after CNV typing to explore whether there are deviations in the major metabolic pathways of cells with different CNV levels. Using the Reactome database and KEGG database metabolic gene sets based on the scMetabolism package, we screened metabolic pathways with the AUCell and ssgsea calculation methods. Finally, we found that only inositol phosphate metabolism showed activity enrichment in both databases and with both calculation methods (Fig 6A-6D). This suggests that Cluster 3 cells may primarily mediate inositol phosphate metabolism reprogramming to maintain their malignant phenotype.

To elucidate the developmental trajectory of tumor cells, we conducted pseudotime analysis. On the developmental trajectory, cells from Cluster 1, Cluster 2, and Cluster 5 were closer to normal cells, while Cluster 6 was primarily located in the middle of the trajectory. Clusters 3 and 4 were positioned at the two ends of the trajectory. Based on the pseudotime analysis, we can infer that Cluster 1, Cluster 2, and Cluster 5 represent early tumor cells, Cluster 6 represents tumor cells with gradually increasing malignancy, and Clusters 3 and 4 represent the two most malignant tumor cells (Fig 6E-6G). For tumor epithelial cells, we extracted the highly variable genes between clusters, and based on the CNV results, Cluster 3 was selected for further analysis.

### Diagnostic and prognostic value of gene pairs

**Fig 7:**
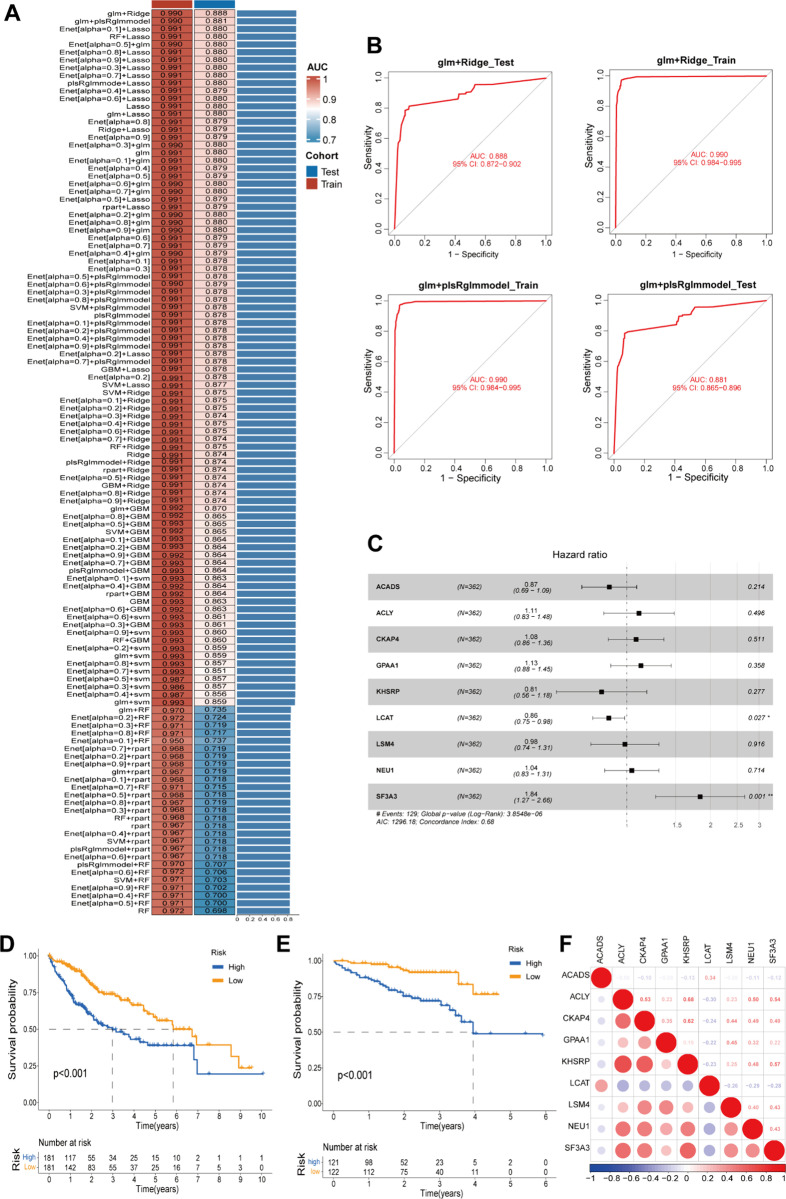
Diagnostic and prognostic models. (A) 117 machine learning algorithms were combined to establish the diagnostic model. **(B)** ROC curves for glm+Ridge and glm + plsRglm model. **(C)** Multivariate COX regression model. **(D)** Kaplan-Meier curves for training set 2. **(E)** Kaplan-Meier curves for validation set 2. **(F)** Coexpression analysis of nine genes.

We extracted the marker genes of Cluster 3 and identified the intersecting genes in the training set to calculate stable reverse gene pairs. Ultimately, we identified seven metabolic reprogramming feature gene pairs: ACADS|GPAA1, ACADS|NEU1, ACLY|LCAT, CKAP4|LCAT, KHSRP|LCAT, LCAT|LSM4, and LCAT|SF3A3. These seven gene pairs were incorporated into a diagnostic model for prediction in the training set, and the model was subsequently validated using a validation set. We found that the prediction results of the glm+Ridge and glm + plsRglm models were the best, with an AUC of 0.990 in the training set, and AUC values of 0.888 and 0.881 in the validation set, respectively (Fig 7A and 7B).

Since binarization results are not suitable for the establishment and use of a prognostic model, we performed univariate and multivariate Cox regression analyses using the nine genes that constitute the gene pairs. In the univariate Cox regression analysis, all genes were found to be statistically significant and thus could serve as prognostic factors. In the model established through multivariate Cox regression analysis, the concordance index (C-index) was 0.68, indicating that the model holds some prognostic predictive value (Fig 7C). We discovered that the risk factor SF3A3 had statistical significance in the multivariate Cox regression model and may become an important prognostic factor for HCC. By stratifying patients into high-risk and low-risk groups based on the median value of the multivariate Cox score, we found that the prognosis of high-risk group patients was significantly worse than that of the low-risk group, as shown by the Kaplan-Meier survival curve (Fig 7D and 7E).

We performed co-expression analysis of the nine model genes in the TCGA cohort and found that the expression of SF3A3 was negatively correlated with ACADS and LCAT, while positively correlated with the other genes (Fig 7F).

### SF3A3 affects the growth and migration ability of hepatocellular carcinoma

**Fig 8:**
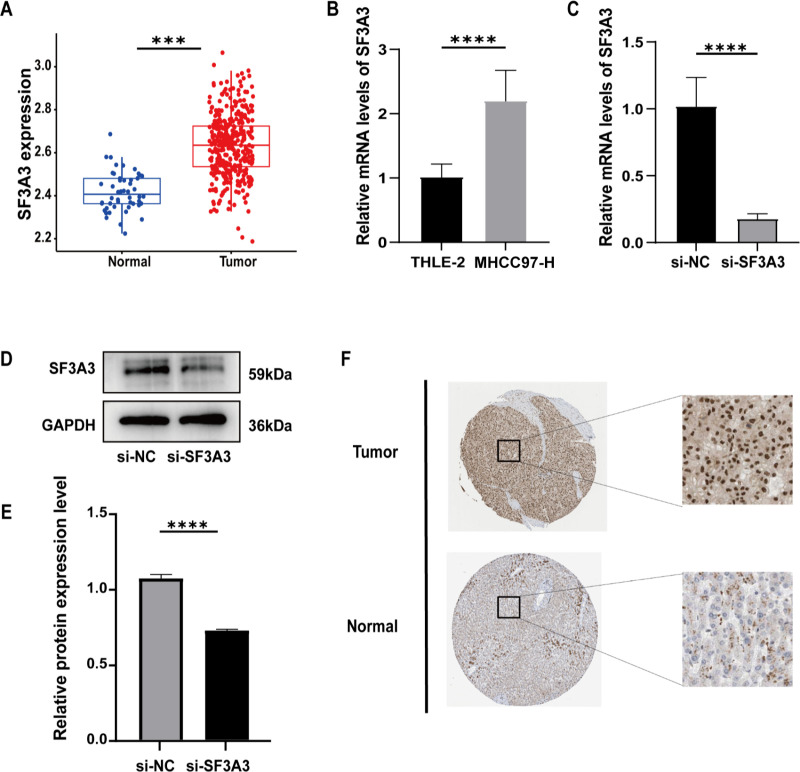
SF3A3 expression analysis and experimental validation. **(A)** Expression of SF3A3 in TCGA database. **(B)** Differences in SF3A3 expression in HCC cells. **(C)** Efficiency of siRNA interference with SF3A3. **(D)** Decreased the expression of SF3A3 protein after interfering with SF3A3. **(E)** Statistical plot of decreased SF3A3 protein expression after SF3A3 interference. **(F)** SF3A3 staining in the HPA database.

Comparative analysis in the TCGA cohort demonstrated markedly higher SF3A3 expression in malignant tissues versus adjacent normal tissues (Fig 8A). qRT-PCR analysis demonstrated significantly higher SF3A3 expression in HCC cell line MHCC97-H relative to normal hepatocyte cell line THLE-2 (Fig 8B). Following siRNA-mediated knockdown of SF3A3 in MHCC97-H cells, qRT-PCR and western blot analysis confirmed the successful knockdown of SF3A3 (Fig 8C-8E). Moreover, immunohistochemical analysis from the HPA database showed that HCC tissues displayed a notably stronger SF3A3 staining intensity compared to normal liver tissues, reinforcing the observation of marked upregulation of SF3A3 in HCC (Fig 8F).

**Fig 9:**
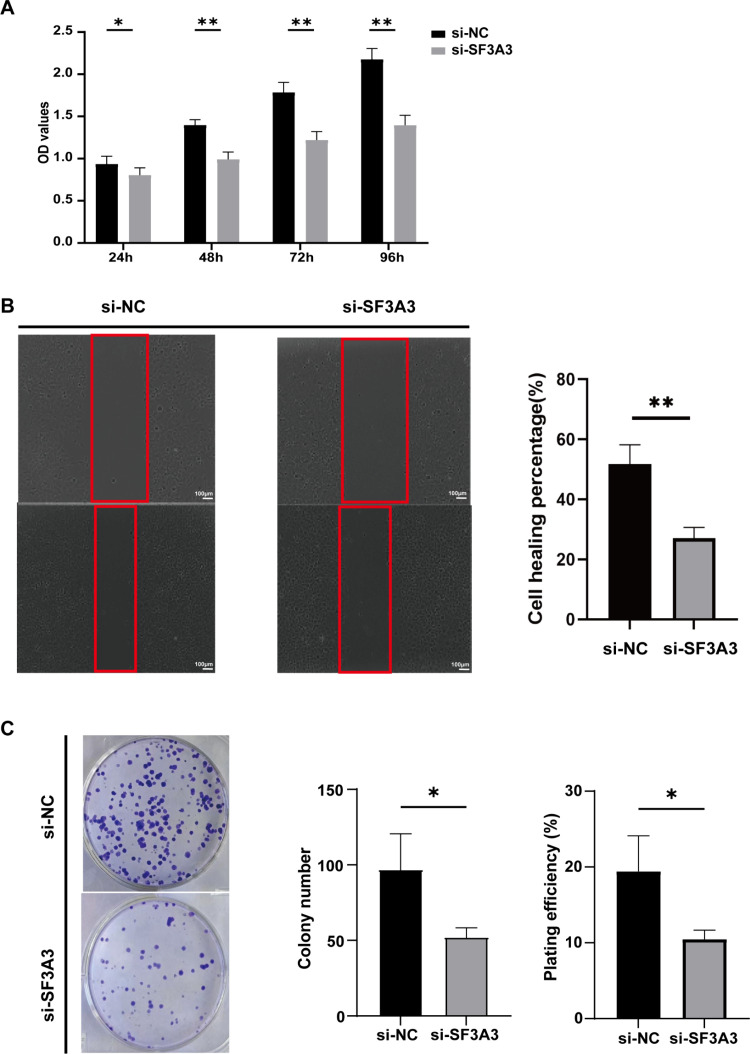
Cell phenotype assays interfering with SF3A3. **(A)** CCK8 assay interfering with SF3A3. **(B)** Cell scratch assay interfering with SF3A3. **(C)** Colony formation assay of interfering SF3A3.

CCK-8 assay demonstrated that the absorbance at 450 nm in the treated group was considerably lower than in the control group, suggesting that disrupting SF3A3 expression considerably diminished the proliferation capability of MHCC97-H cells (Fig 9A). Using a scratch test, we found that silencing SF3A3 notably hindered the migration and invasion of MHCC97-H cells (Fig 9B), which indicates that SF3A3 might play a role in the EMT in HCC. The malignancy of a tumor can often be assessed by its tumor-initiating potential. To evaluate how SF3A3 interference affected the tumor-initiating ability of MHCC97-H cells, we conducted a colony formation assay. Our results showed that after disrupting SF3A3 expression, the colonies formed by MHCC97-H cells were both fewer in number and smaller in size when compared to the control group (Fig 9C). This implies that knockdown of SF3A3 effectively reduces the cancer cells’ capacity to initiate tumors.

### Silencing SF3A3 could down-regulate the PI3K/AKT signaling pathway and reduce the expression of EMT markers

**Fig 10:**
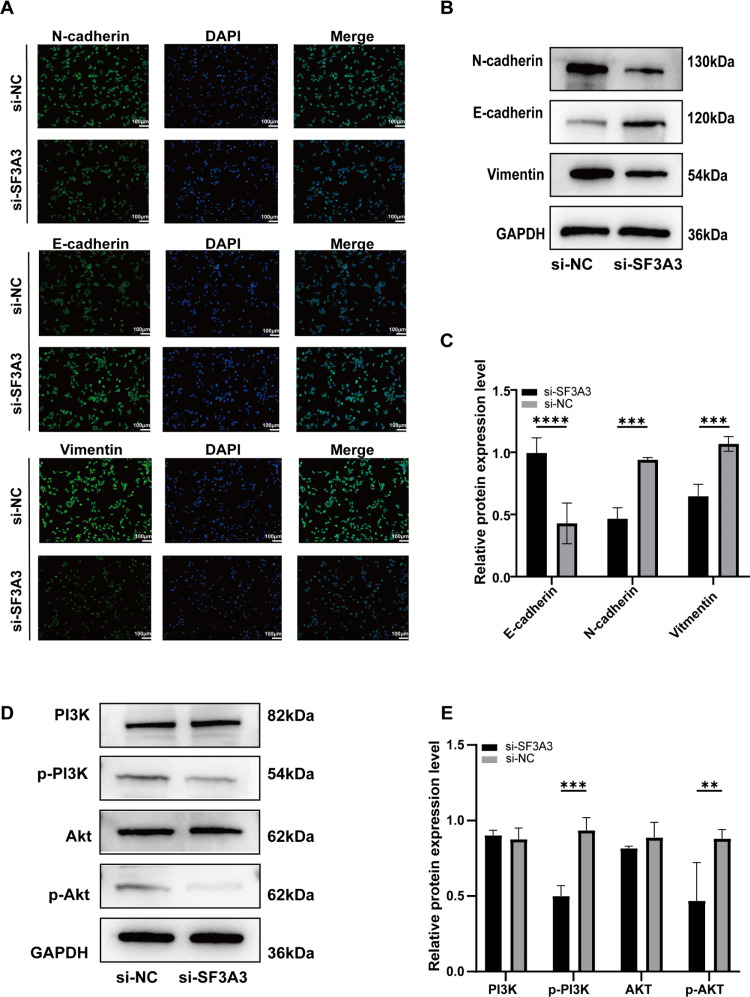
Immunofluorescence and western blot analysis. **(A)** Immunofluorescence of E-cadherin, N-cadherin and Vimentin. **(B)** Western blot of E-cadherin, N-cadherin and Vimentin. **(C)** Western blot statistics of E-cadherin, N-cadherin and Vimentin. **(D)** Western blot of PI3K/AKT signaling pathway. **(E)** Western blot statistical plot of PI3K/AKT signaling pathway.

By immunofluorescence, we found that the EMT marker E-cadherin was increased, while N-cadherin and Vimentin were decreased after silencing SF3A3 (Fig 10A). To further confirm our fluorescence results, we performed western blot experiment. Western blot results showed that the expression of EMT-related proteins was decreased after silencing SF3A3 (Fig 10B and 10C). Furthermore, western blot analysis of PI3K/AKT signaling pathway showed that the protein expression levels of p-PI3K and p-AKT were down-regulated after silencing SF3A3 (Fig 10D and 10E).

## Discussion

HCC is one of the most common and deadly malignancies worldwide, with a high incidence and mortality rate [25]. Despite significant advances in cancer research, the prognosis for HCC patients remains poor, mainly due to the lack of effective early diagnostic methods and targeted therapies [26]. Therefore, there is an urgent need to identify novel biomarkers and therapeutic targets for HCC.

In this study, by using scRNA-seq to identify and annotate different cell subsets in HCC tissues, we found that mononuclear phagocytes, epithelial cells, endothelial cells, NK/T cells, and B cells subsets play different roles in the tumor microenvironment. Among the epithelial cell subsets, tumor-derived epithelial cells exhibit higher levels of copy number variation, which may be associated with the malignant progression of tumors [27,28]. Using cluster analysis, we further revealed differences in CNV levels between these cell subsets. The cluster with the highest level of CNV change is Cluster 3, which may represent the most malignant tumor cell subset.

GO enrichment analysis of cell subsets revealed their roles in immune response, cell communication and metabolic pathways. For example, the role of mononuclear phagocytes for immune cell adhesion and recognition, NK/T cells are activated in immune response, B cells produce antibodies, and endothelial cells are crucial in angiogenesis. These findings highlight the critical role of various types of cells within the tumor tissue in maintaining the HCC tumor microenvironment and provide potential new strategies for targeted therapy [29]. Cell communication and metabolic analyses revealed complex interactions and metabolic reprogramming among HCC cells. Communication between epithelial and endothelial cells may be critical for the formation of the tumor microenvironment and immune escape [30–32]. Metabolic reprogramming of epithelial cells is more obvious, which may be related to the proliferation and invasion of tumor cells.

Through pseudotime analysis, we uncovered the malignant developmental trajectory of HCC cell subgroups. The Cluster 3 subgroup exhibited the highest malignant potential, consistent with its CNV levels. Additionally, cells in Cluster 3 displayed increased phosphoinositide metabolism, potentially linked to the metabolic reprogramming of phosphoinositides. These findings provide new insights into the molecular mechanisms of HCC and potential therapeutic targets.

The diagnostic and prognostic value analysis of gene pairs further confirmed the importance of metabolic reprogramming signature genes in HCC [19,33]. We calculated gene pairs based on features related to metabolic reprogramming and built HCC diagnostic models using 117 machine learning methods. Among them, glm+Ridge and glm + plsRglmmodel had the best prediction performance and could effectively distinguish tumor samples from normal samples. Prognostic analysis showed that SF3A3 could be used as an important prognostic factor. In addition, SF3A3 expression is associated with the malignant degree and survival rate of HCC patients, suggesting that it may become a new biomarker for individualized treatment of HCC.

SF3A3, along with SF3A1 and SF3A2, comprises the SF3a complex of the human U2 snRNP, which is essential for spliceosome formation [34]. SF3a complex facilitates the conversion of the 15S U2 snRNP into the fully functional 17S U2 snRNP, a critical step in pre-mRNA splicing and spliceosome maturation [35–37]. Elevated expression of spliceosome components ensures precise pre-mRNA splicing, supporting cancer cell growth and survival [38].Previous study have shown that the upregulation of SF3A3 expression by its promoter hypomethylation, thereby mediating bladder cancer progression [39]. Notably, SF3A3 has been shown to interact with MYC in breast cancer models, where translational modulation of SF3A3 induces metabolic reprogramming and acquisition of stem-like properties, ultimately potentiating MYC-driven oncogenesis in vivo [40]. Besides, CircSCAP interacts with SF3A3 to inhibit the malignancy of non-small cell lung cancer by activating p53 signaling [41]. These findings highlight the multifaceted roles of SF3A3 in cancer. Our experimental results showed that knockdown of SF3A3 significantly reduced the proliferation and migration, suppressed PI3K/AKT signaling pathway activity, and downregulated EMT marker expression in HCC cell, which indicated that silencing SF3A3 could affect the PI3K/AKT signaling pathway and inhibit EMT in HCC.

Summarily, our study revealed the complex network of HCC tumor microenvironment and its further clustering in terms of metabolic reprogramming, and identified SF3A3, a prognostic factor in HCC. However, in present study, SF3A3’s clinical pathological analysis and animal experiments were not performed, so the mechanism of action of SF3A3 is still unclear. The molecular mechanisms underlying SF3A3-mediated metabolic reprogramming in HCC requires further investigation.

## Conclusion

Our study delineates the metabolic heterogeneity in HCC epithelial cells and identifies SF3A3 as a critical prognostic determinant. Interfering with SF3A3 can affect the PI3K/AKT signaling pathway and EMT of liver cancer cells, and then affect the growth of cells.

## Supporting information

S1 Fig Single cell clustering dendrogram. (PDF)

S2 Fig The relationship between betweenness centrality and number of clusters. (PDF)

S3 Fig Selecting the number of clusters by the elbow plot. (PDF)

S1 File Raw images of western blot. (PDF)

S2 File Data of statistical graphs. (XLSX)
