# Supplementary figures and images for: Exploring the level of metabolic reprogramming and the role of prognostic factor SF3A3 in hepatocellular carcinoma through integrated single-cell landscape analysis
Source: PLoS One. 2025 May 27;20(5):e0323559. doi: 10.1371/journal.pone.0323559 (PMC12111341; doi:10.1371/journal.pone.0323559)

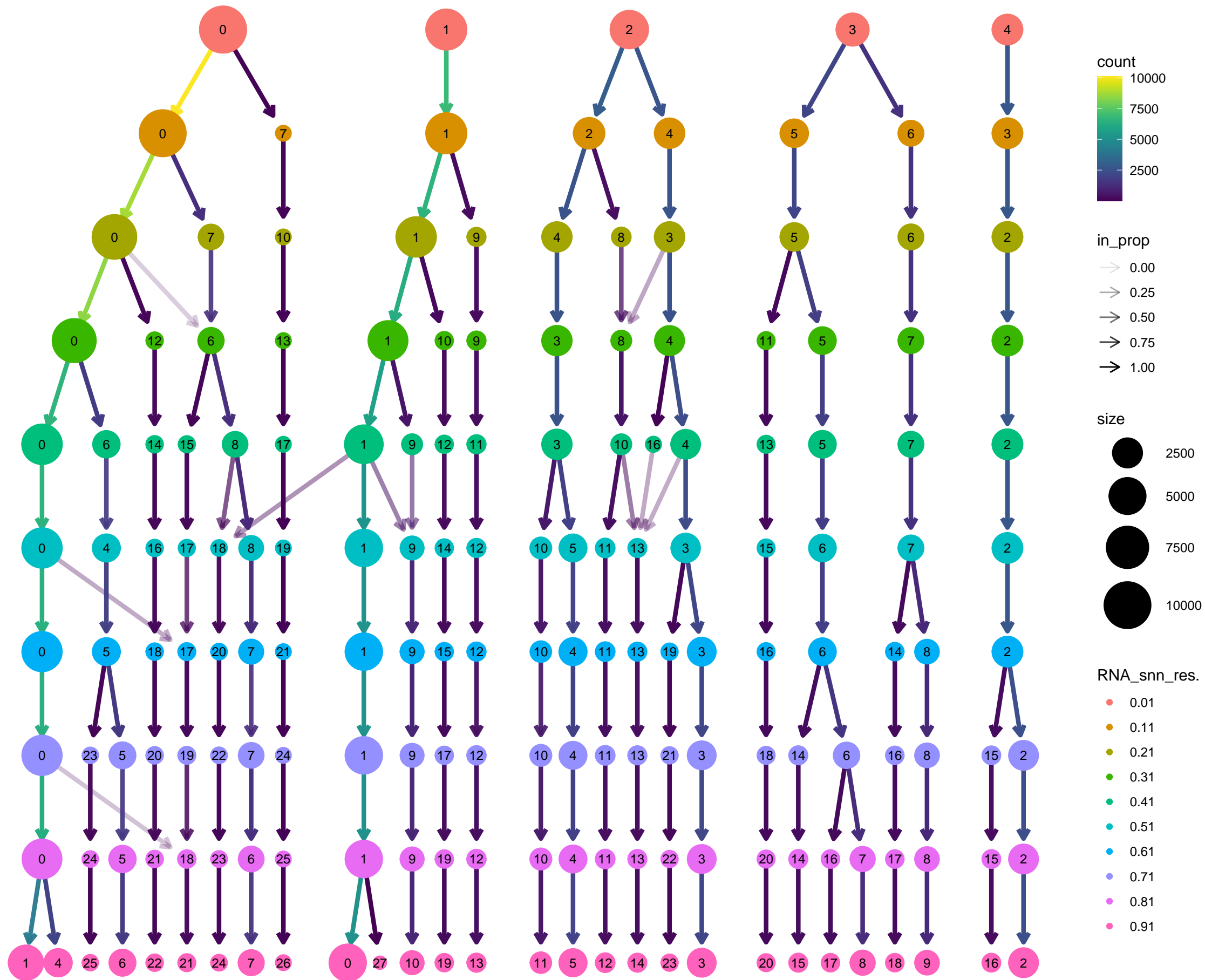

Supplement: S1 Fig — (PDF) [file pone.0323559.s001.pdf]

# Betweenness vs Number of Clusters

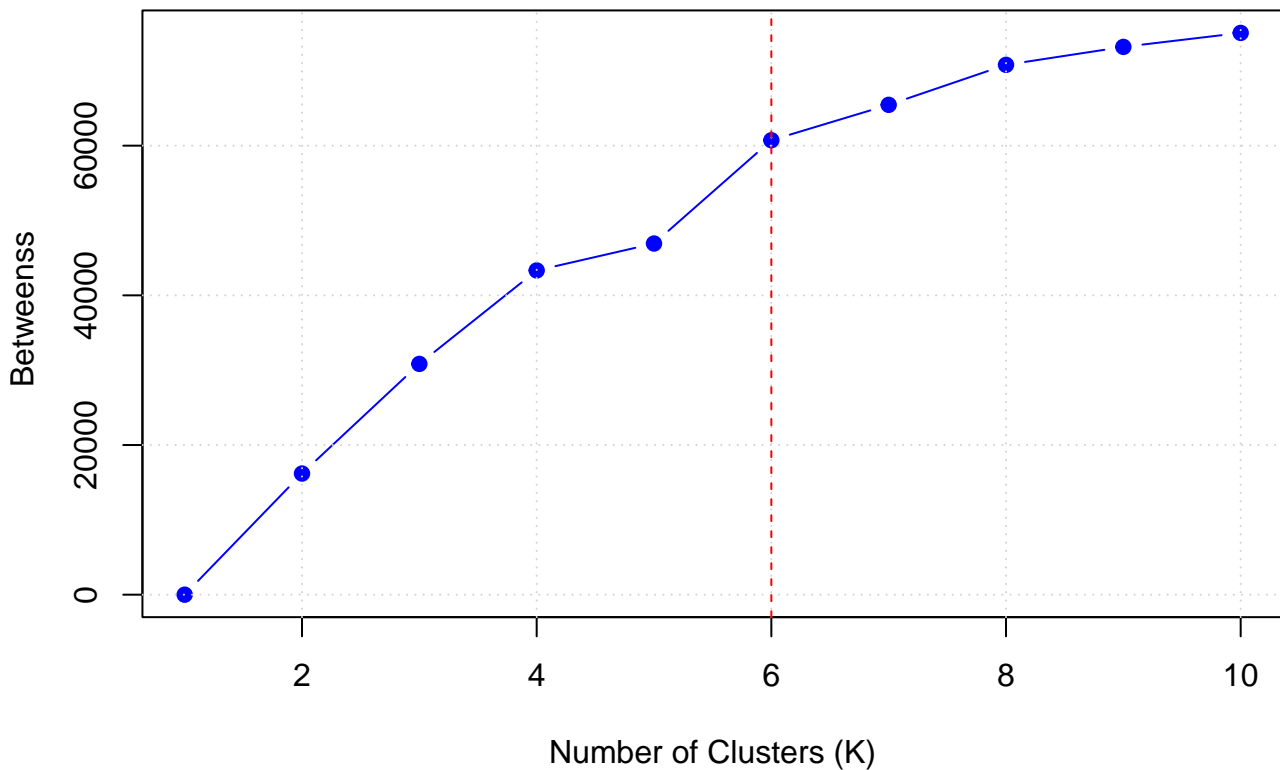

Supplement: S2 Fig — (PDF) [file pone.0323559.s002.pdf]

Elbow Method

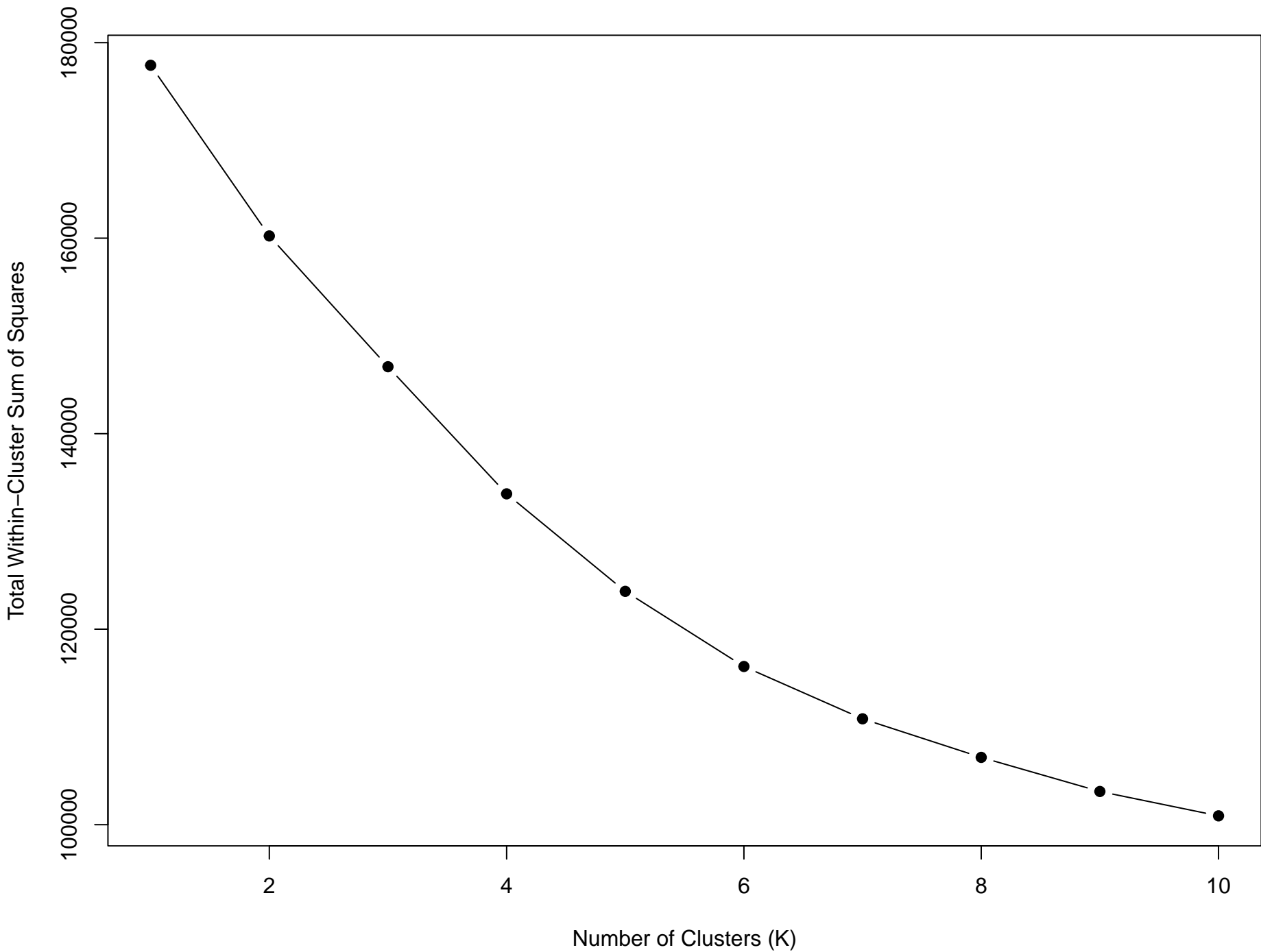

Supplement: S3 Fig — (PDF) [file pone.0323559.s003.pdf]

E-Cadherin

1:

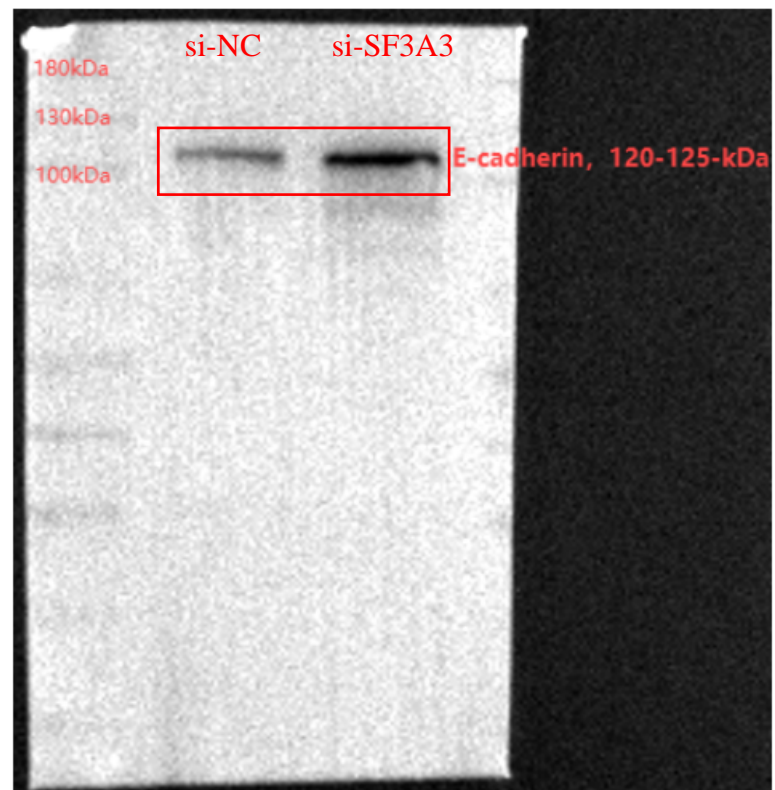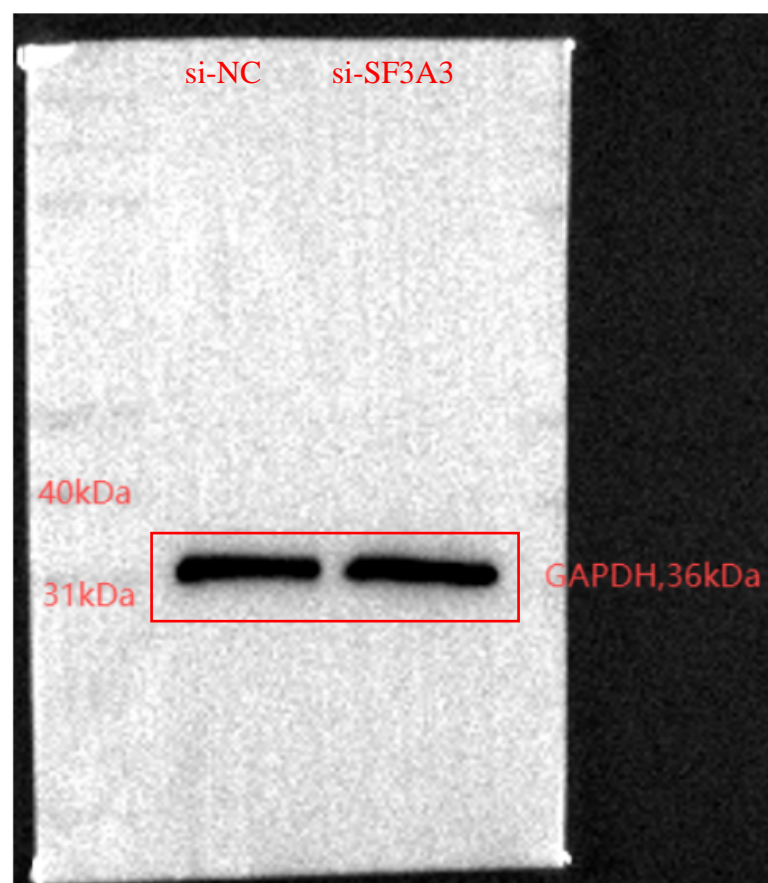

2:

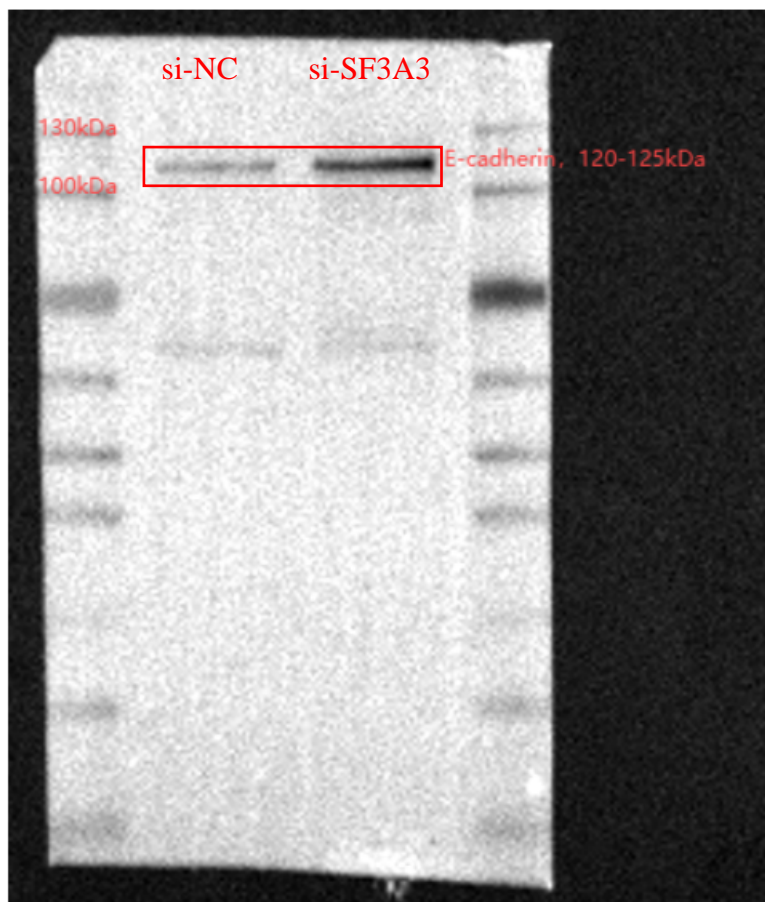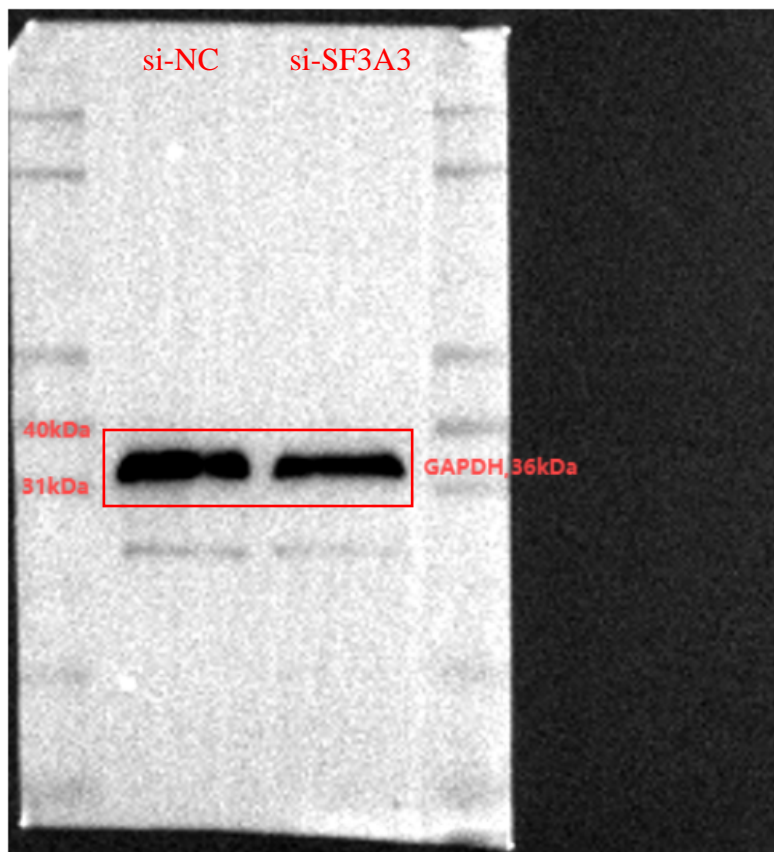

3:

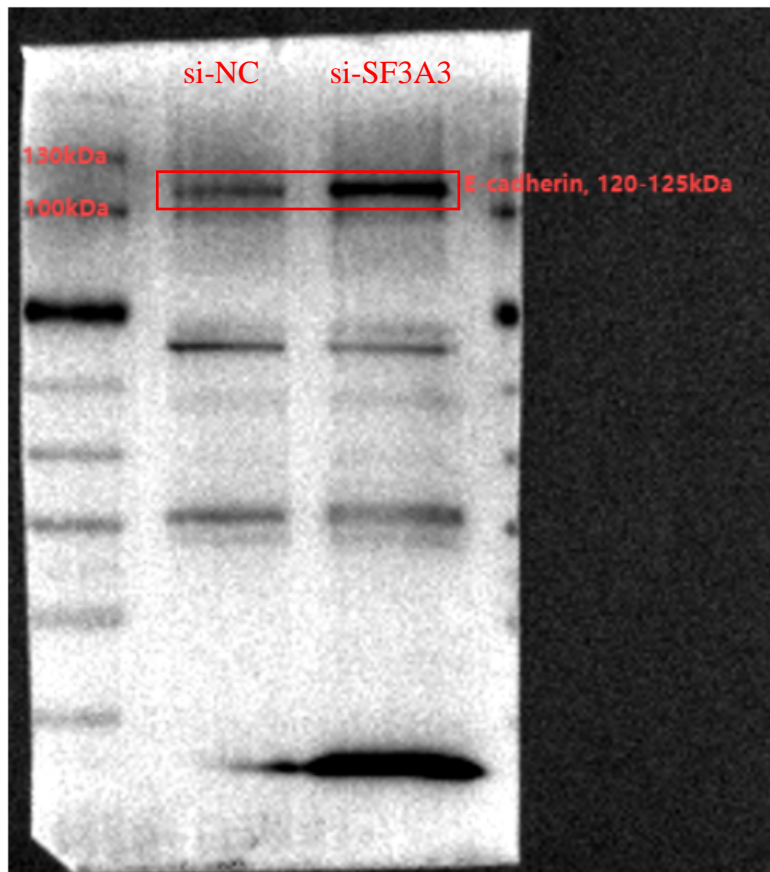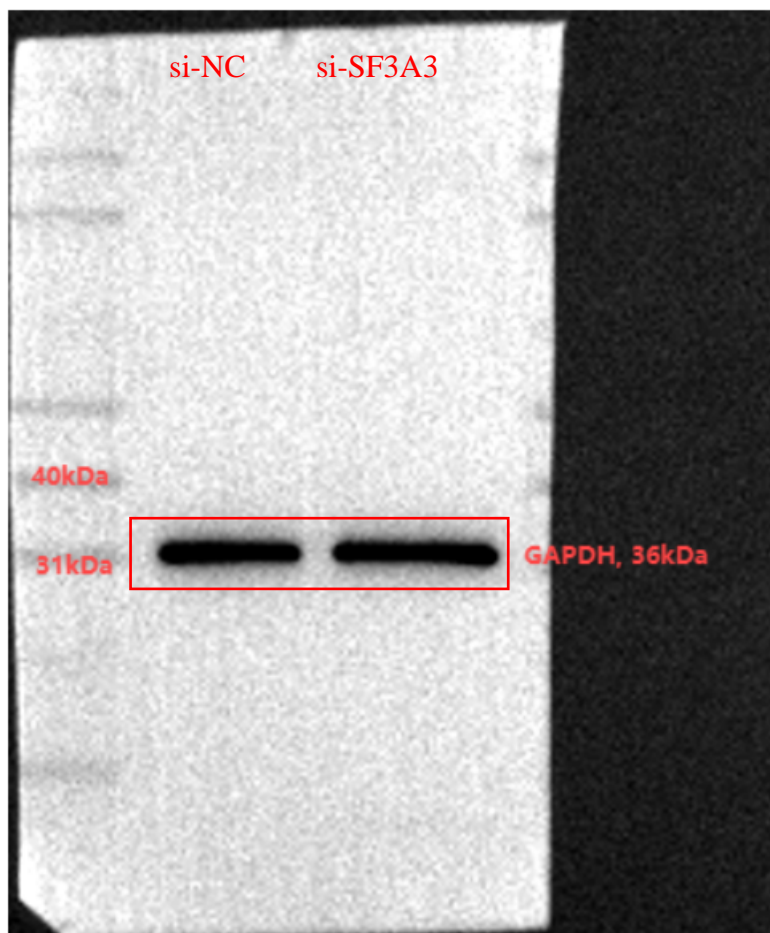

N-Cadherin

1:

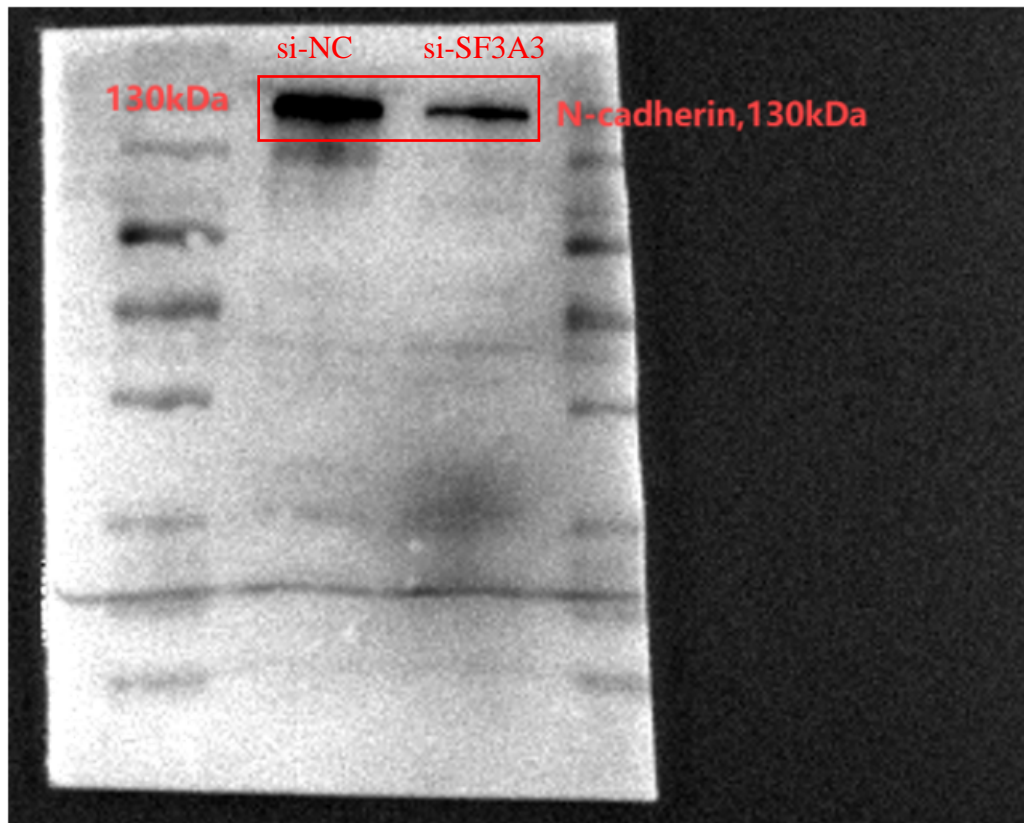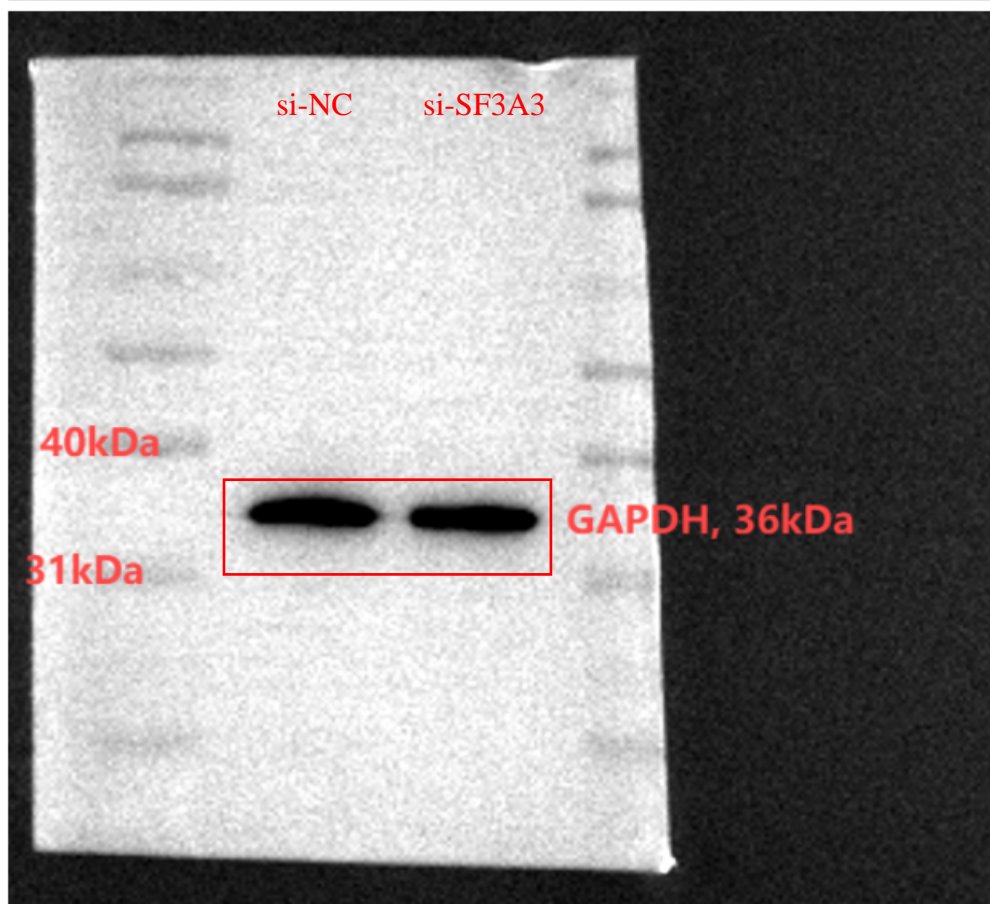

2:

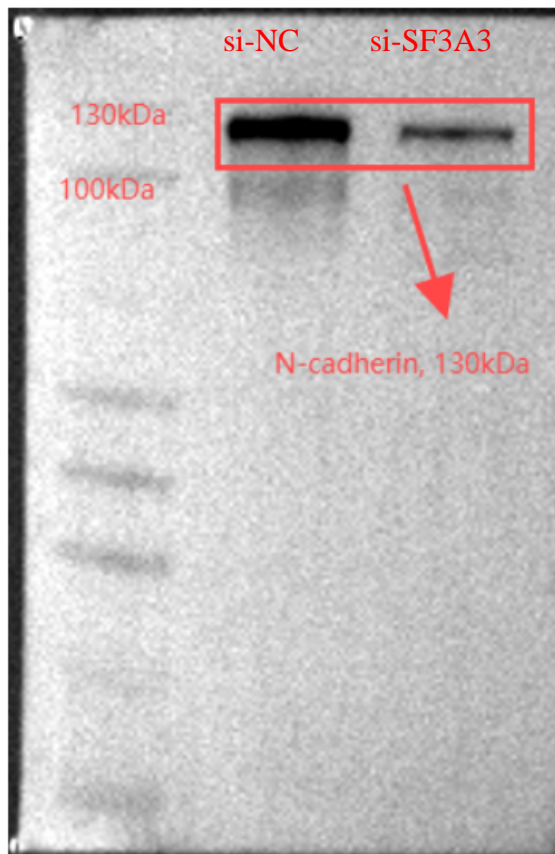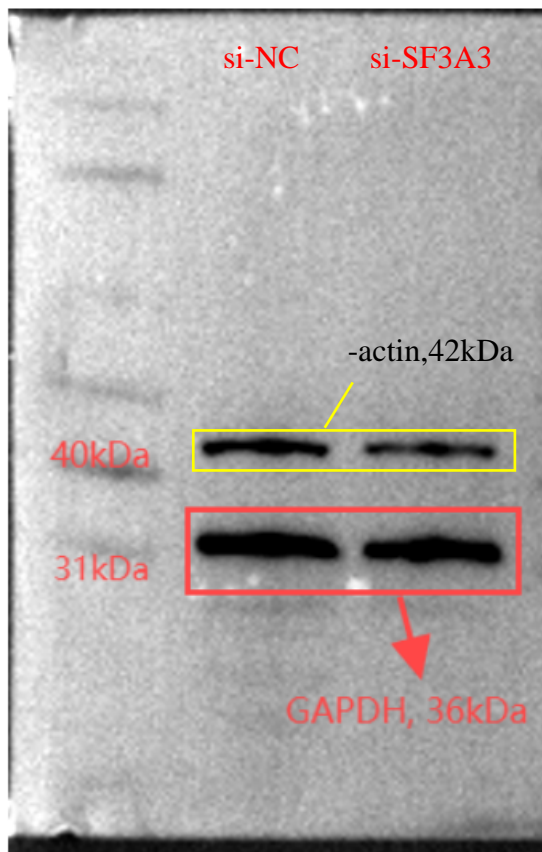

3:

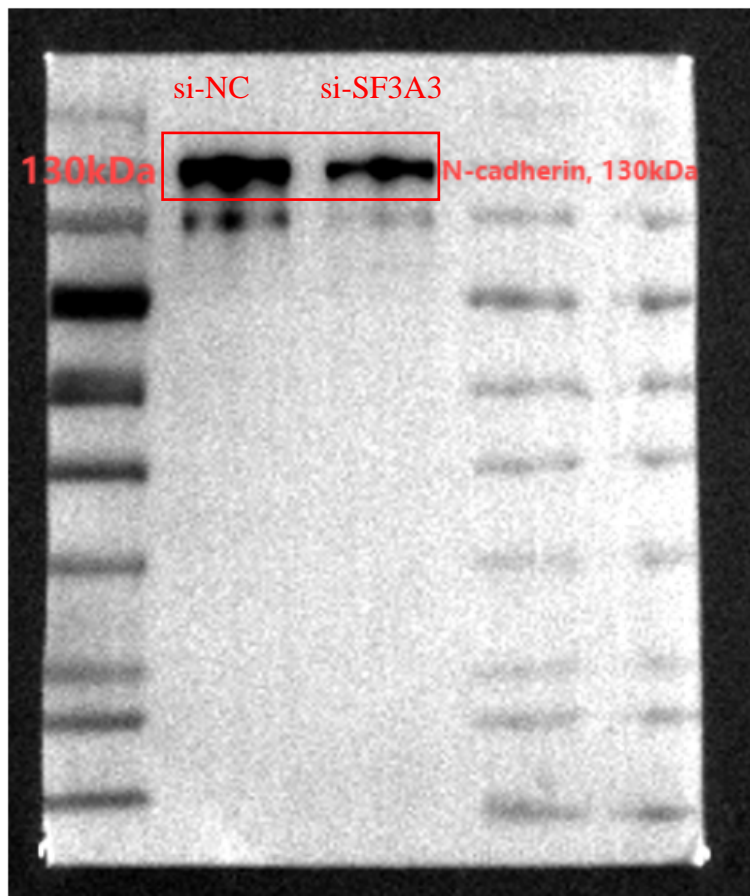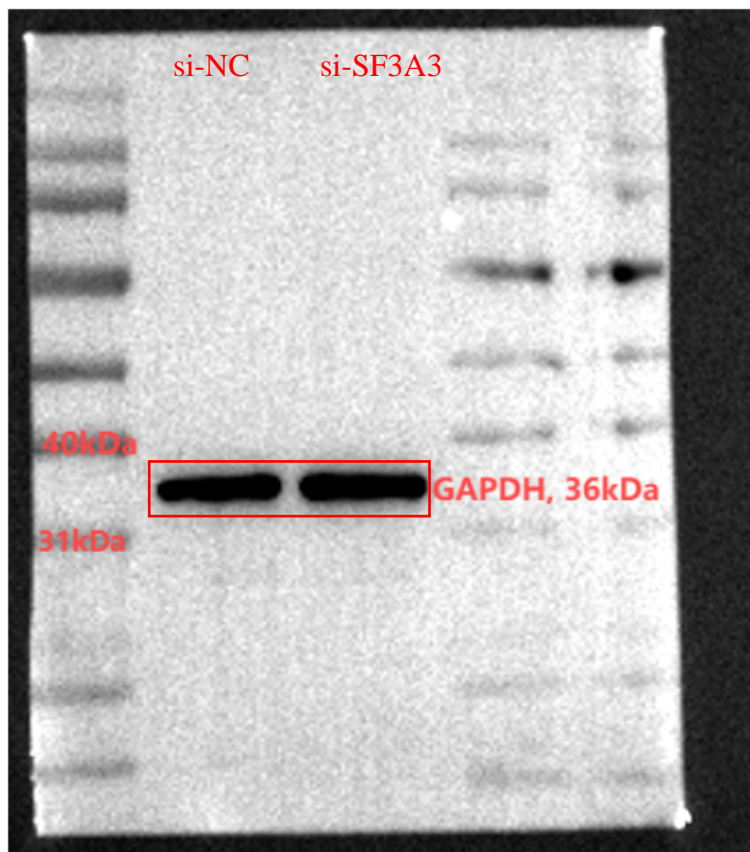

Vimentin:

1:

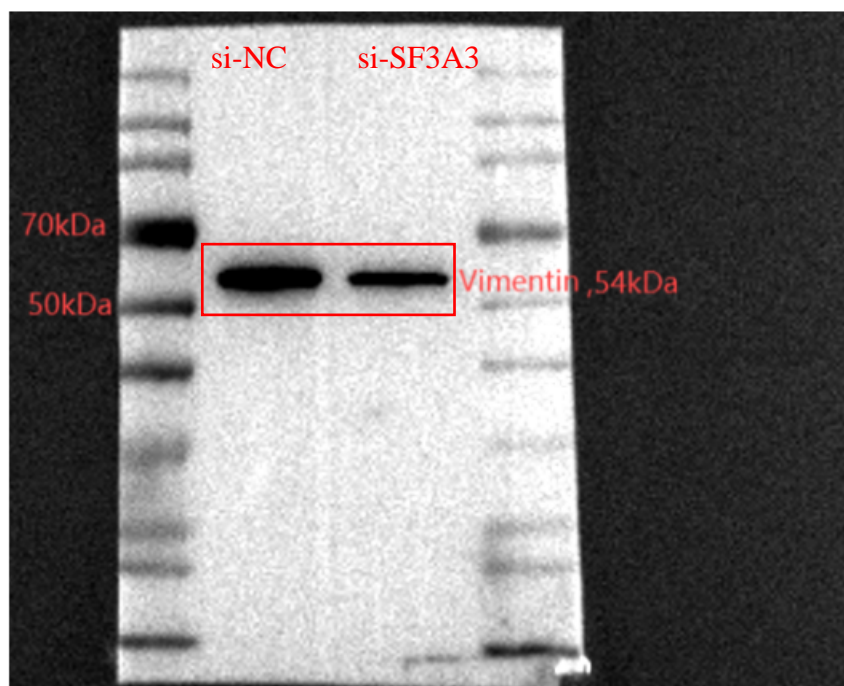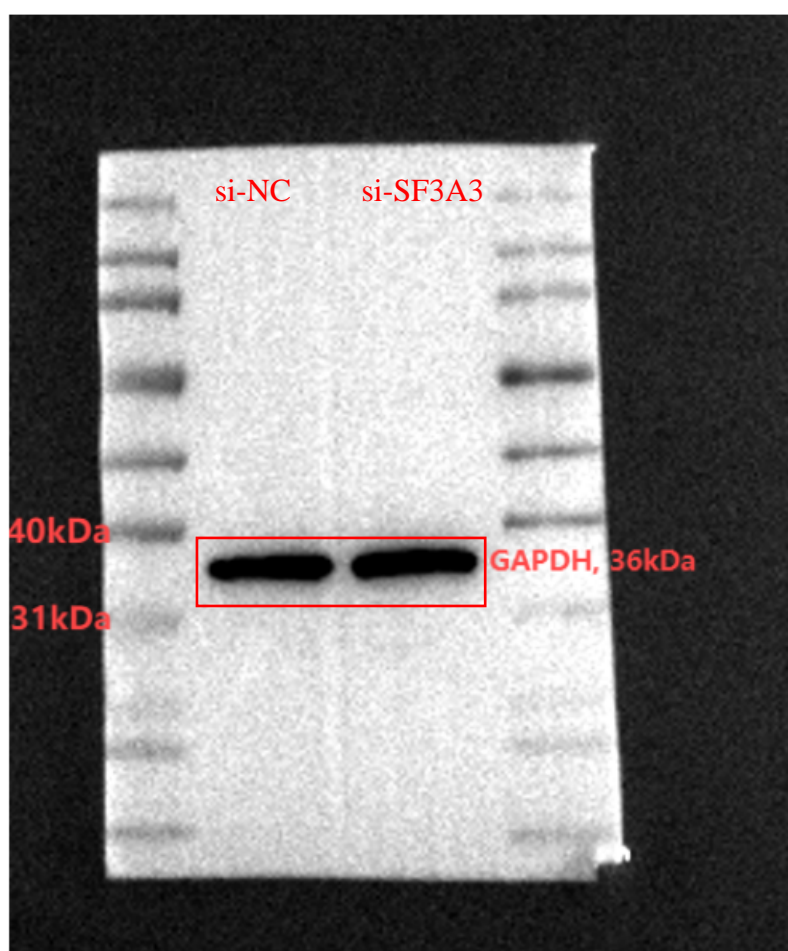

2:

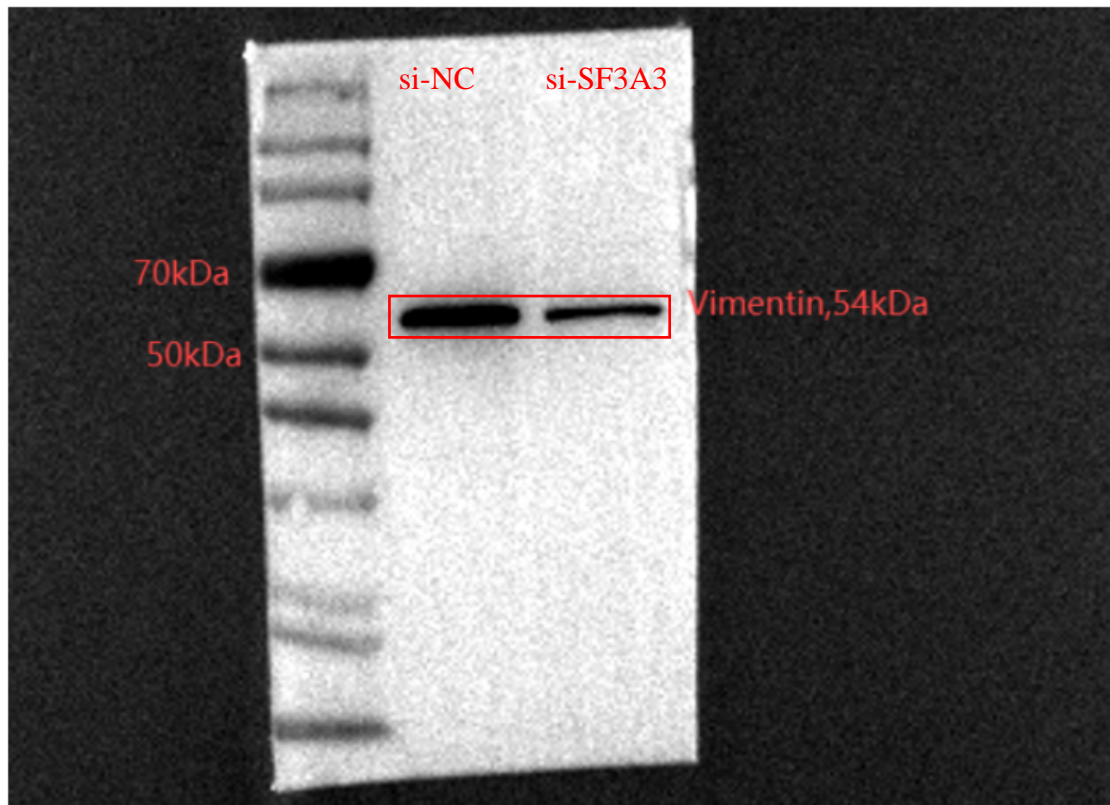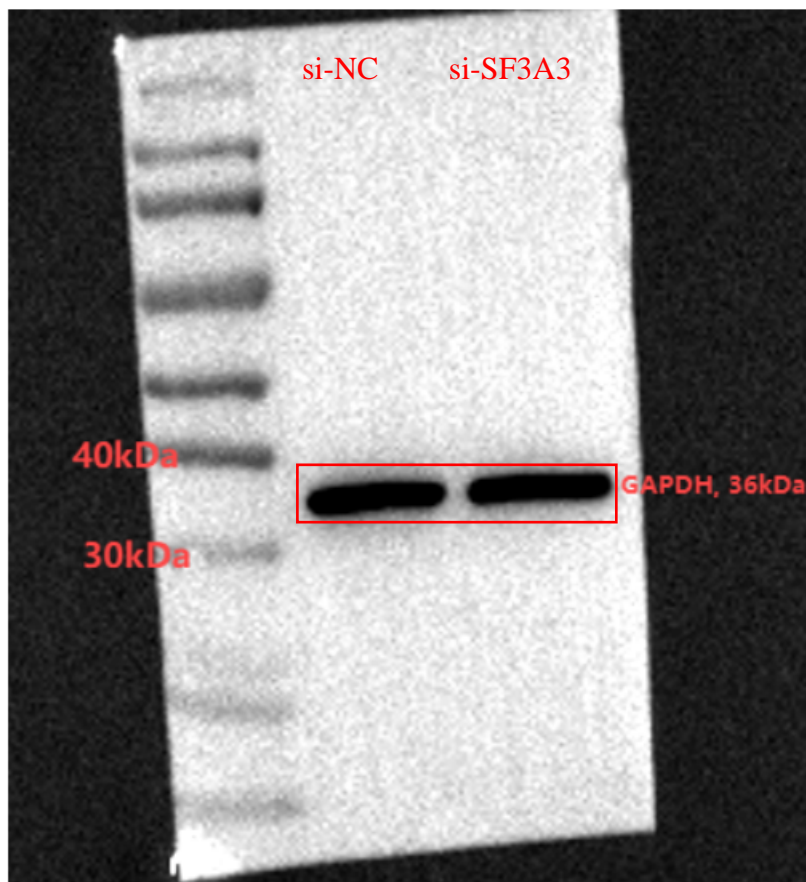

3:

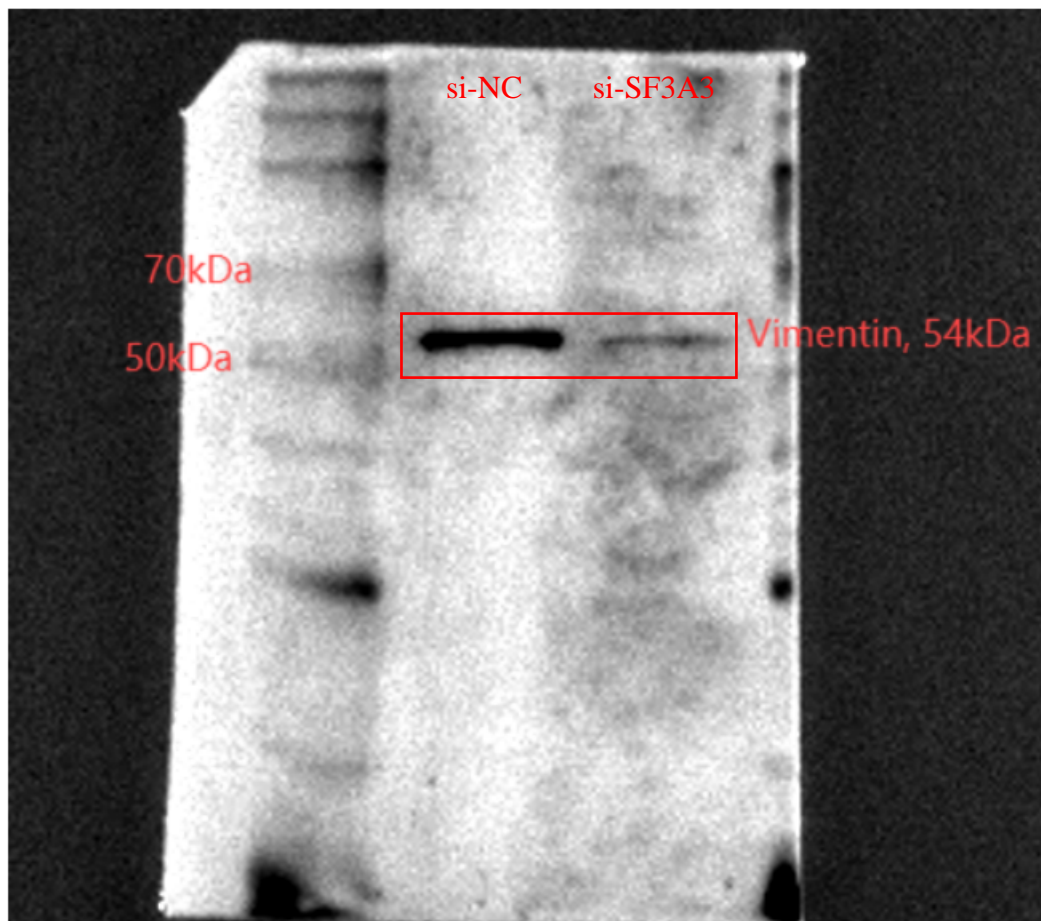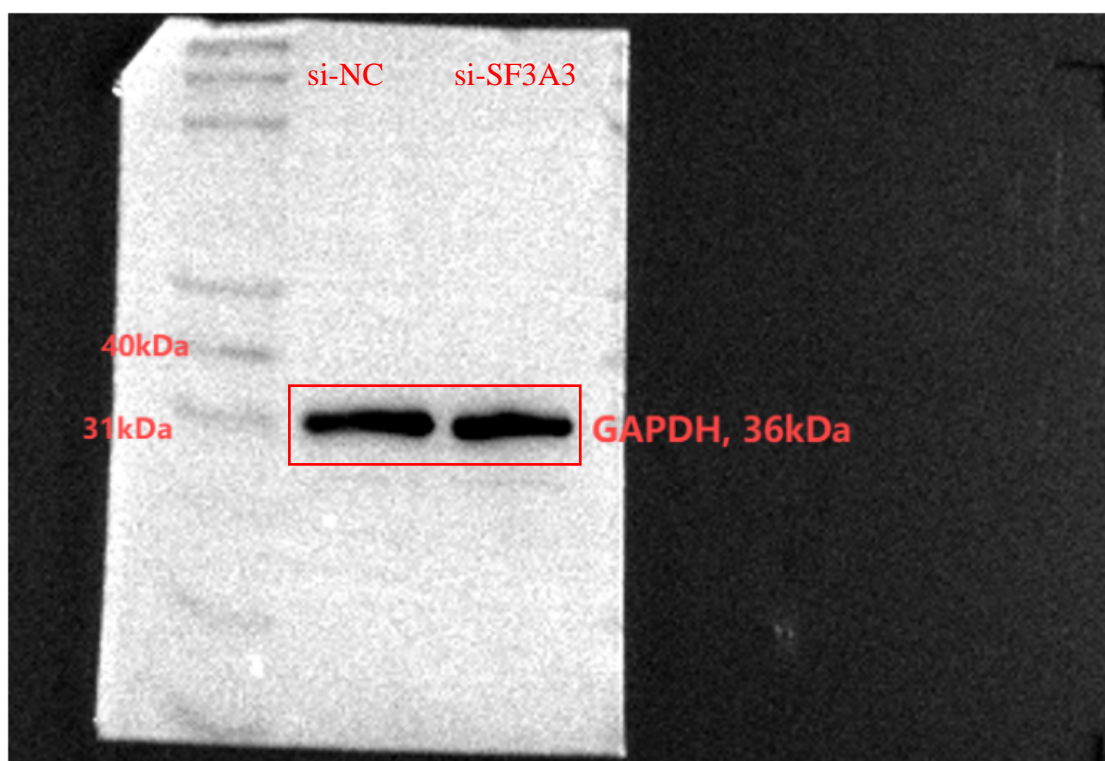

PI3K:

1:

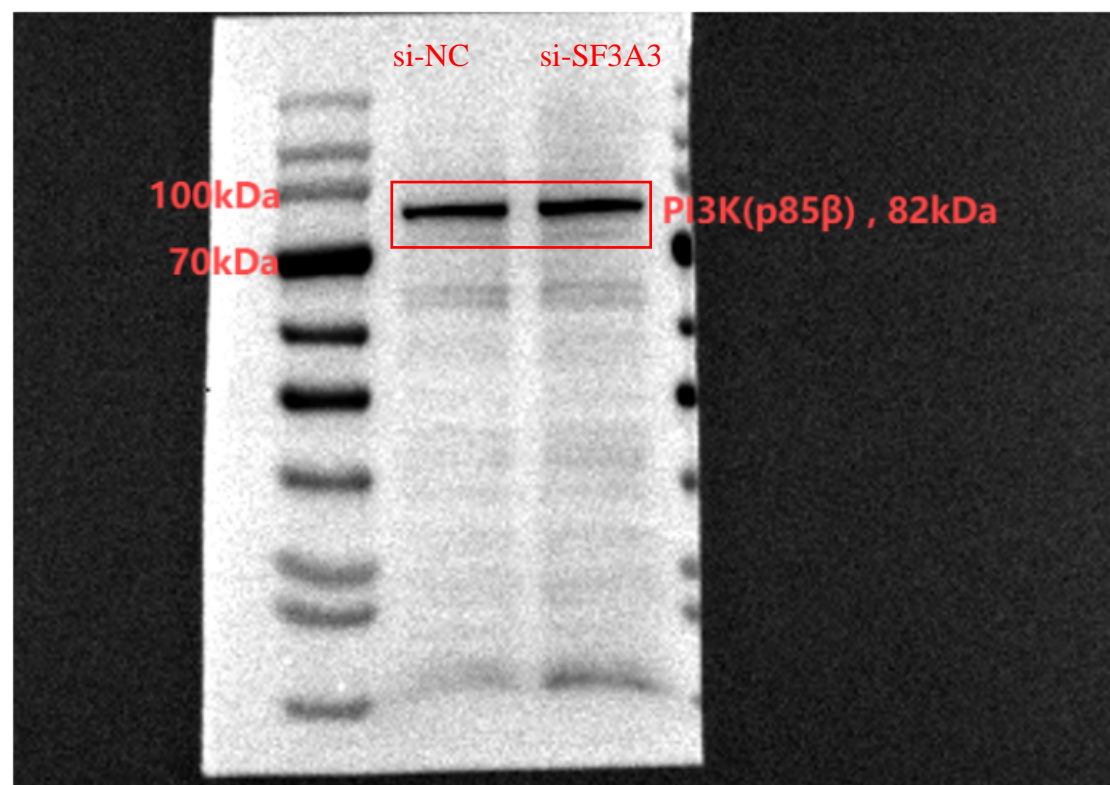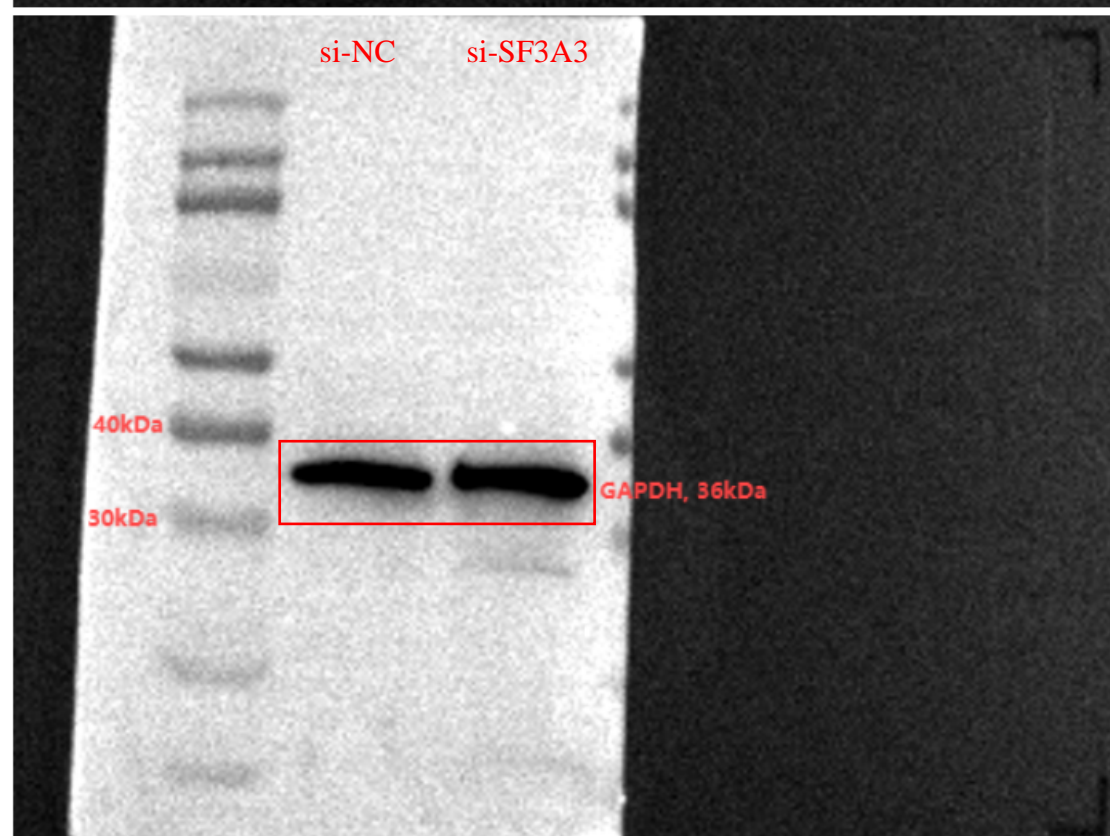

2:

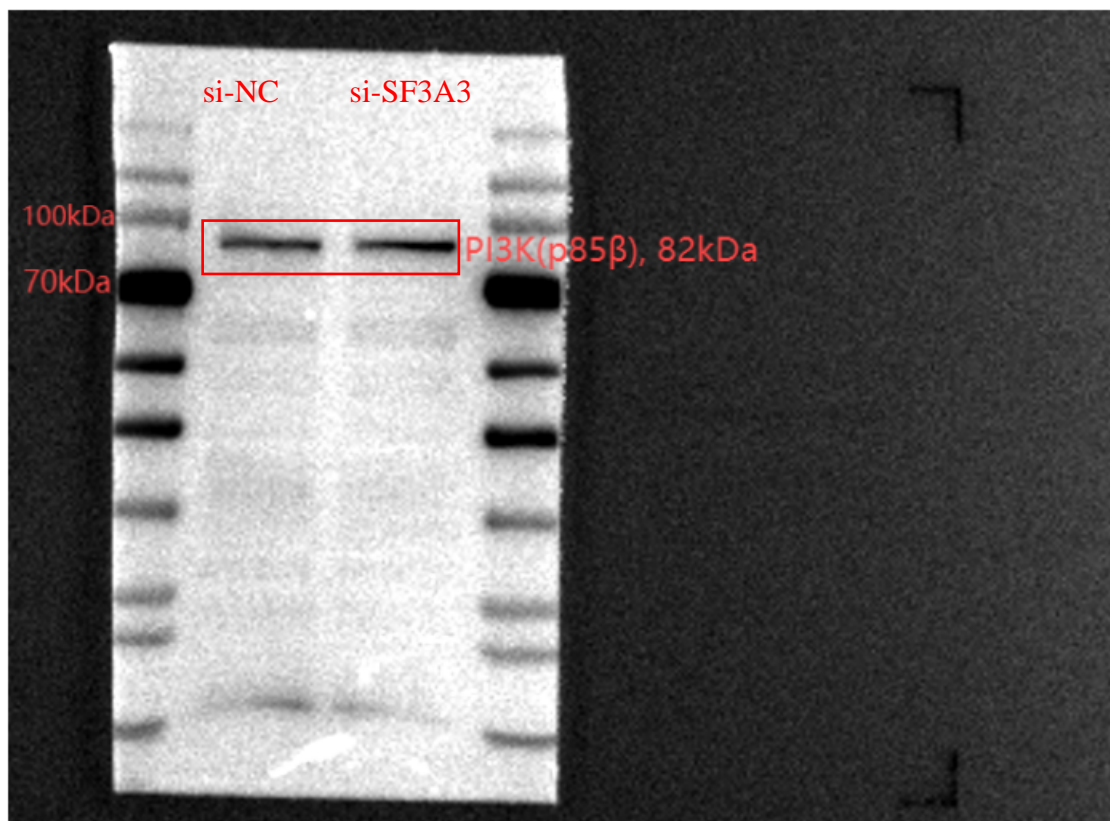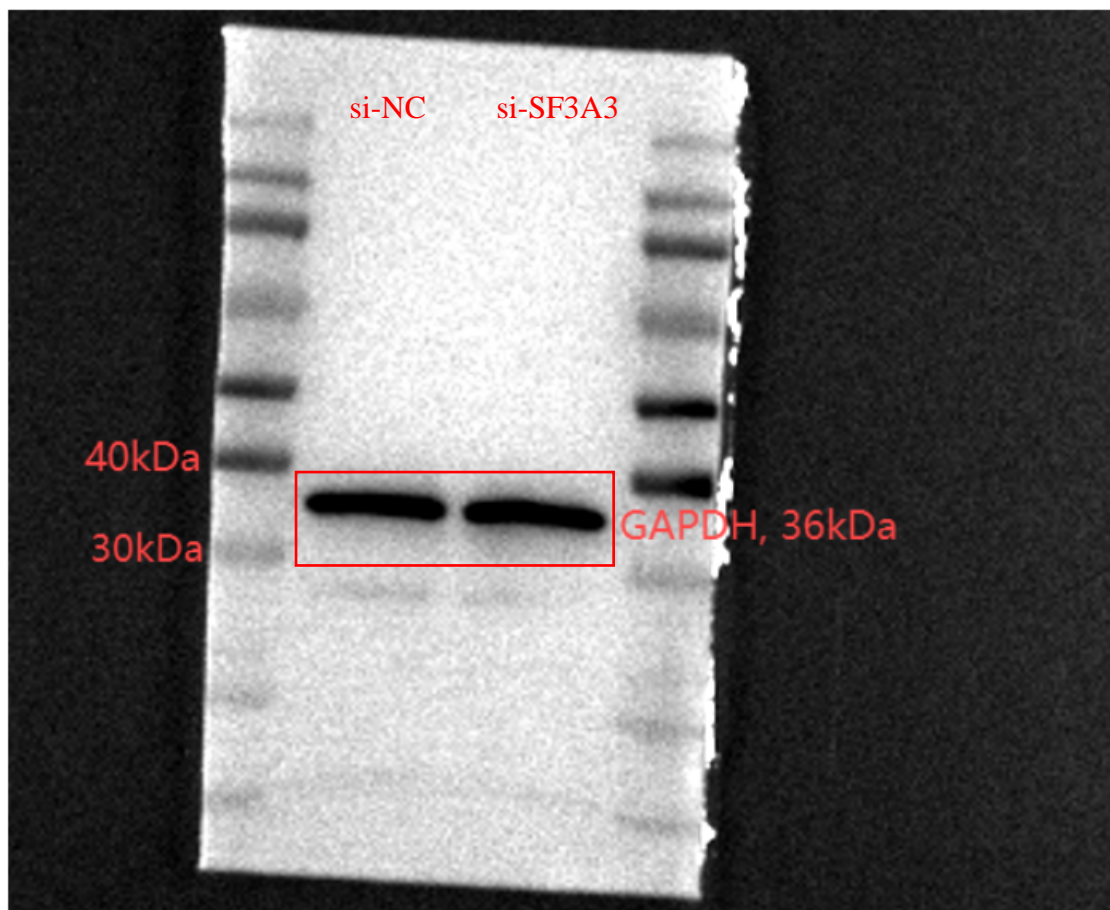

3:

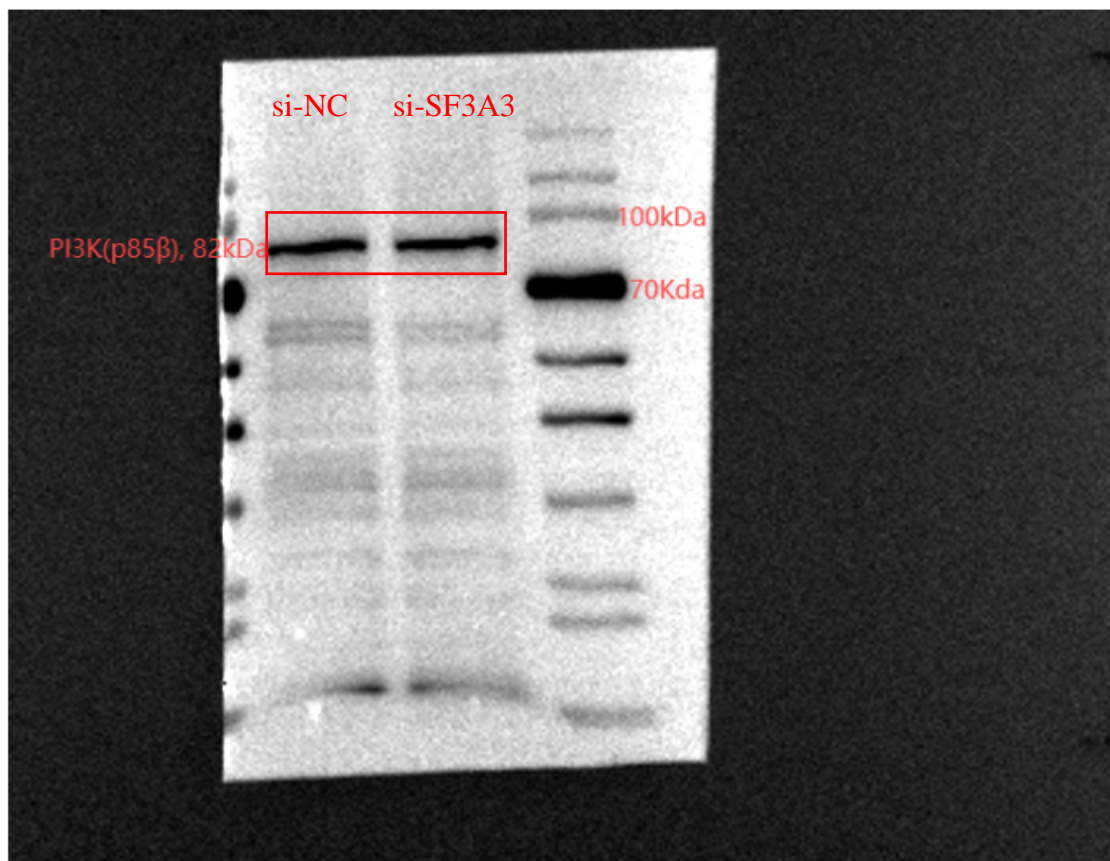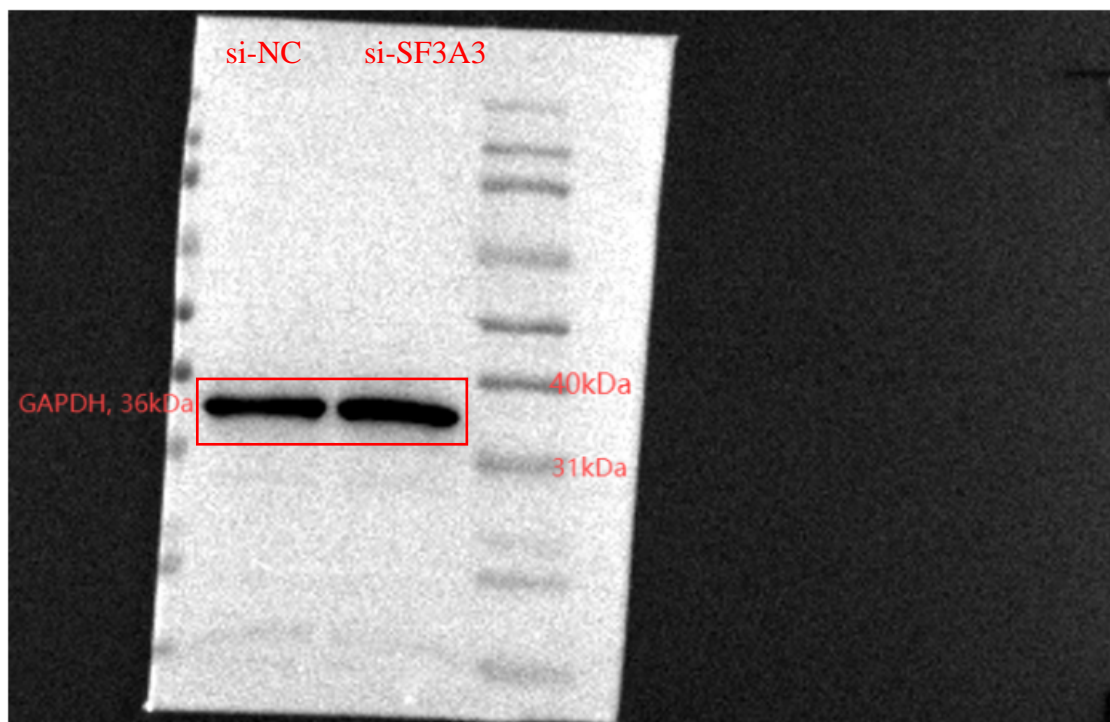

p-PI3K:

1:

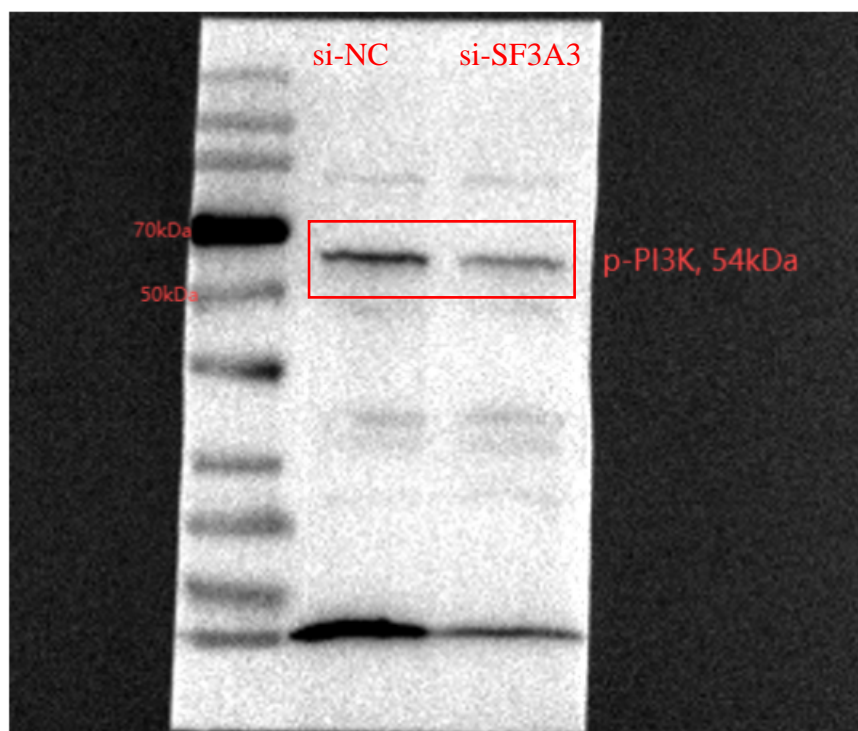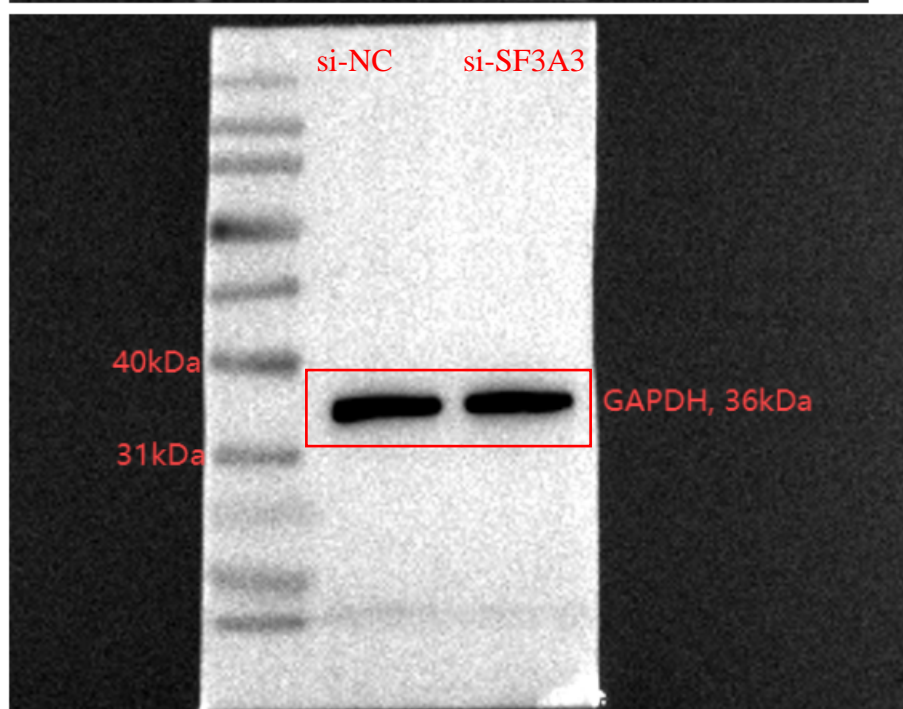

2:

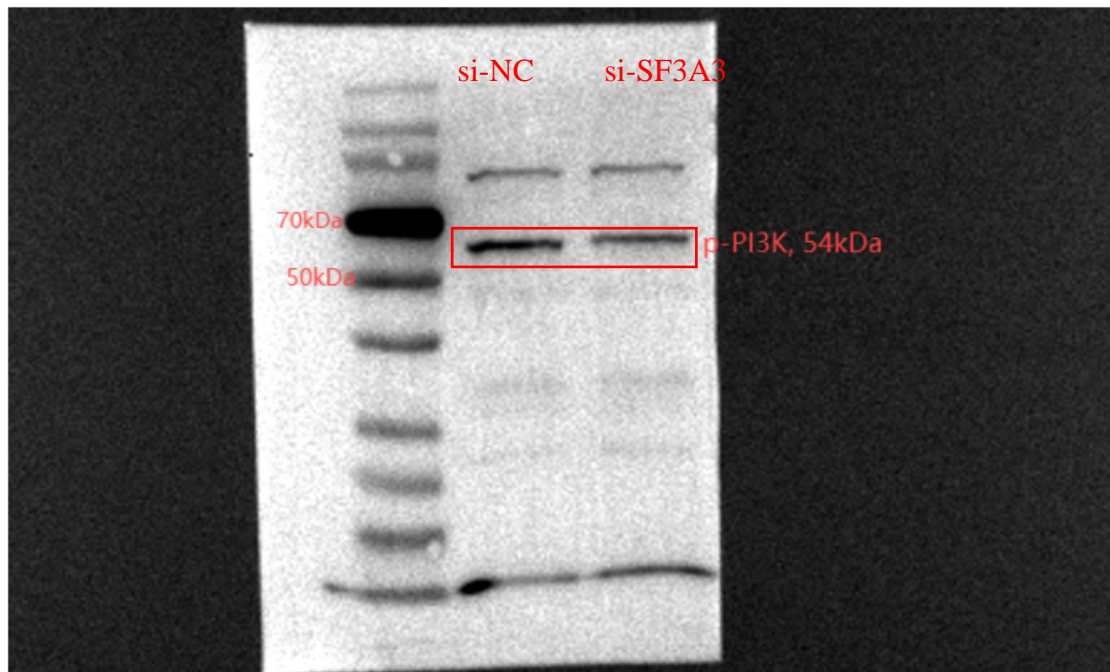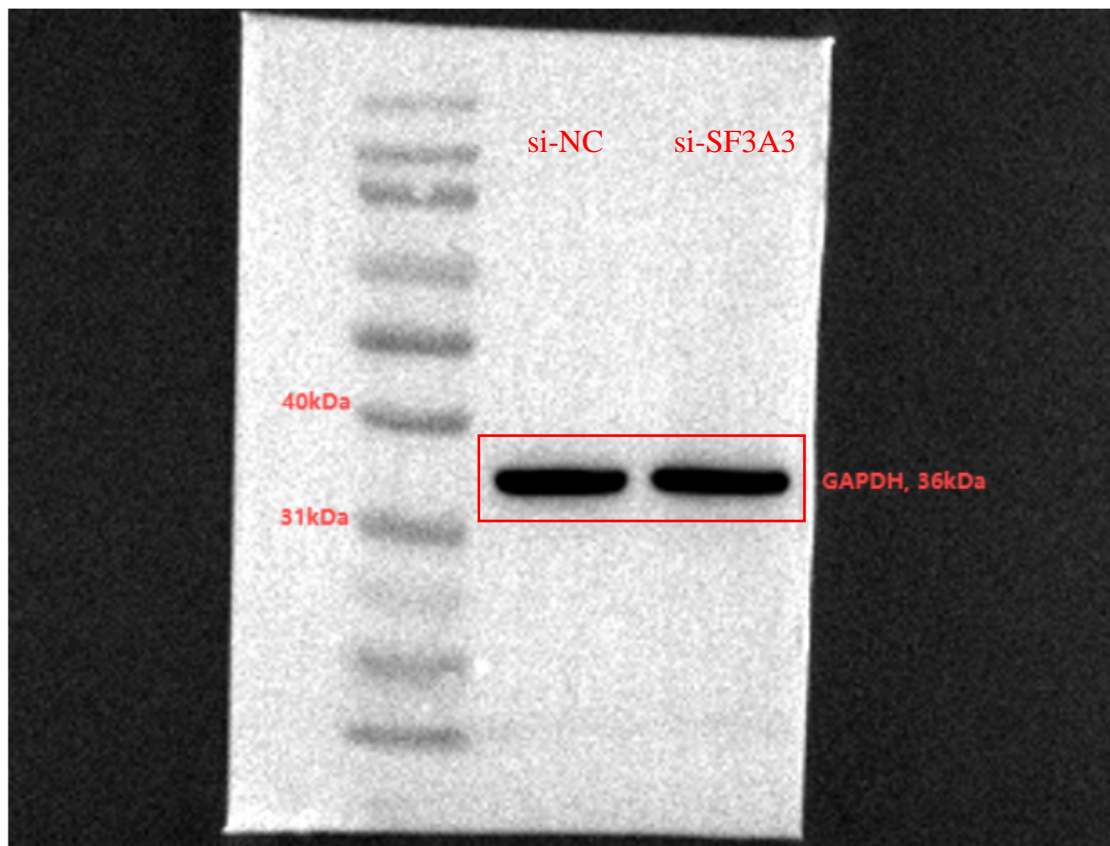

3:

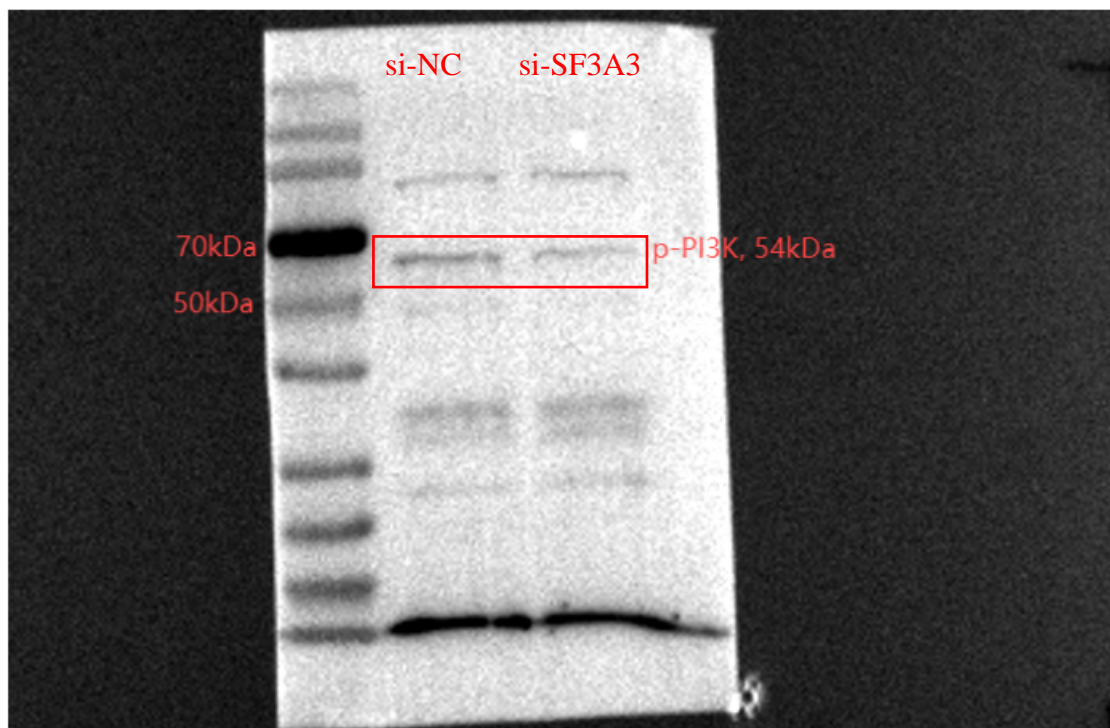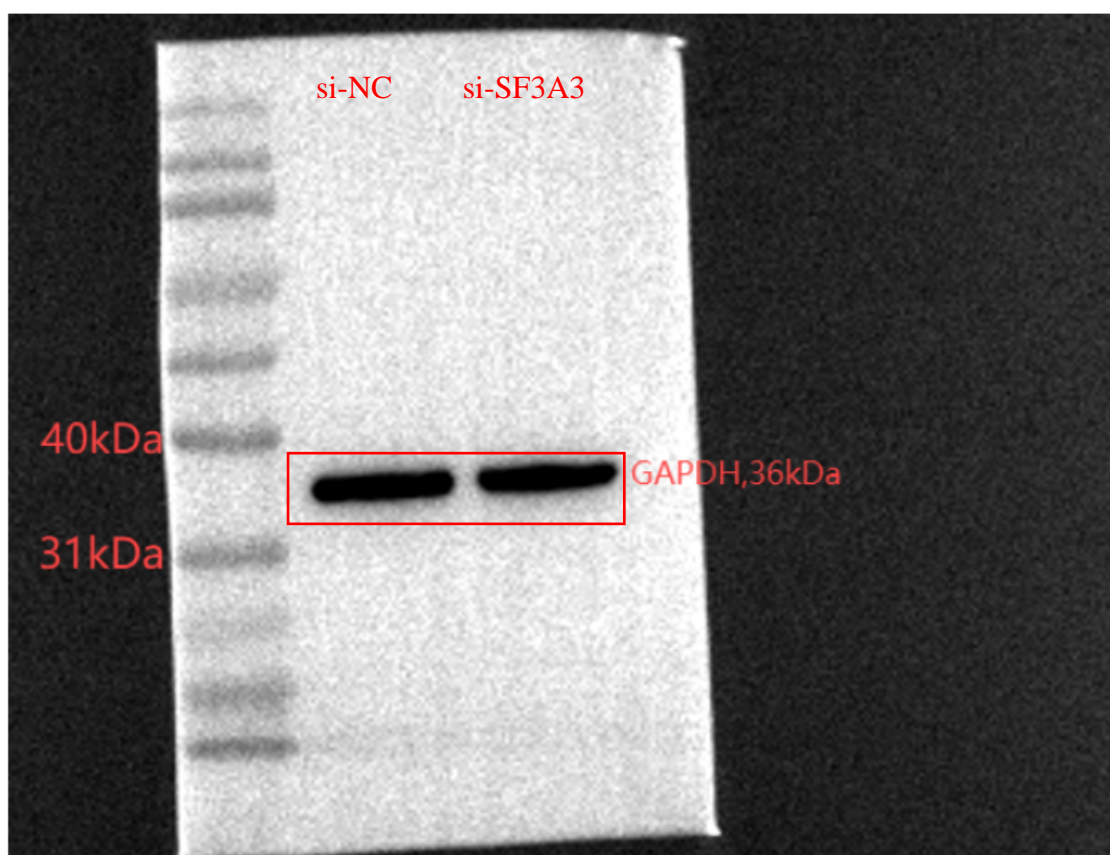

AKT:

1:

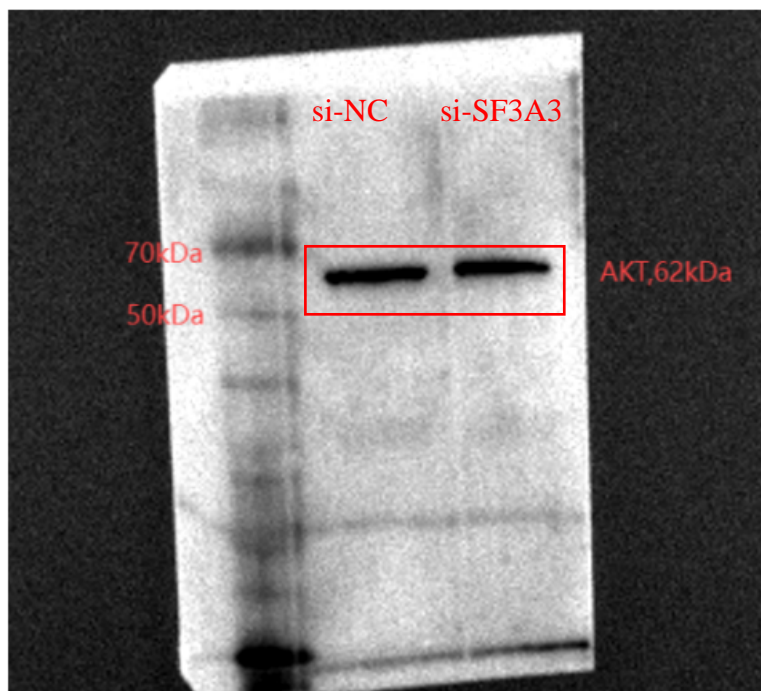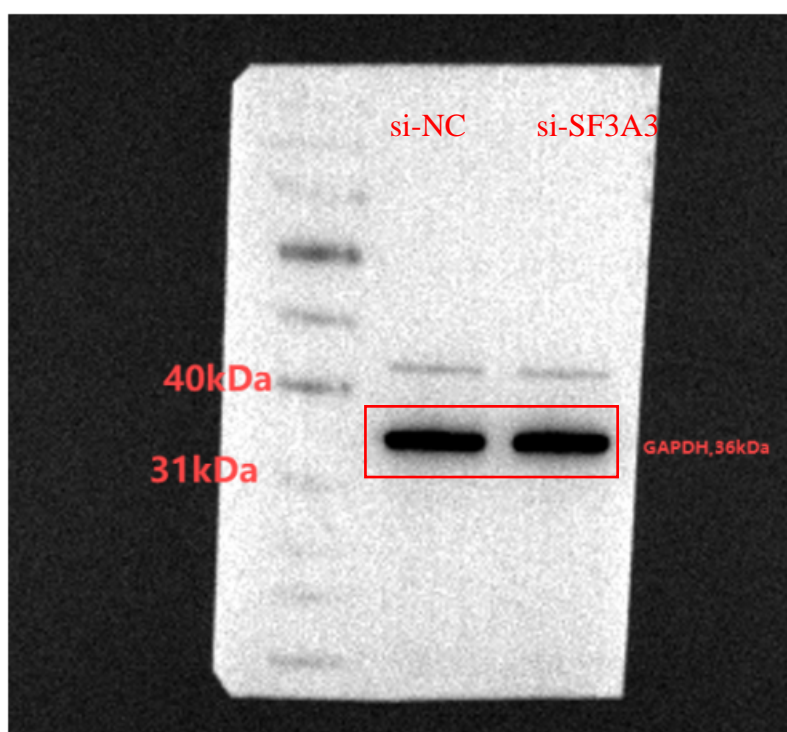

2:

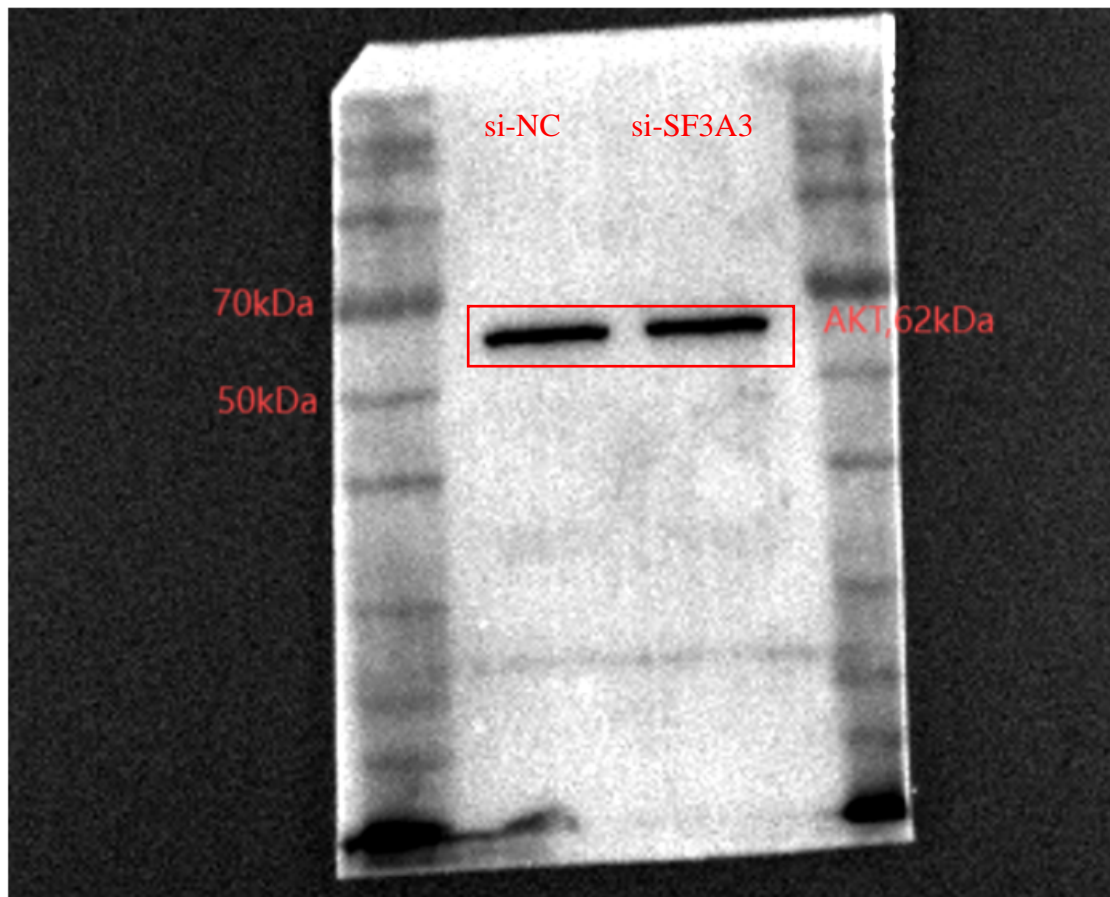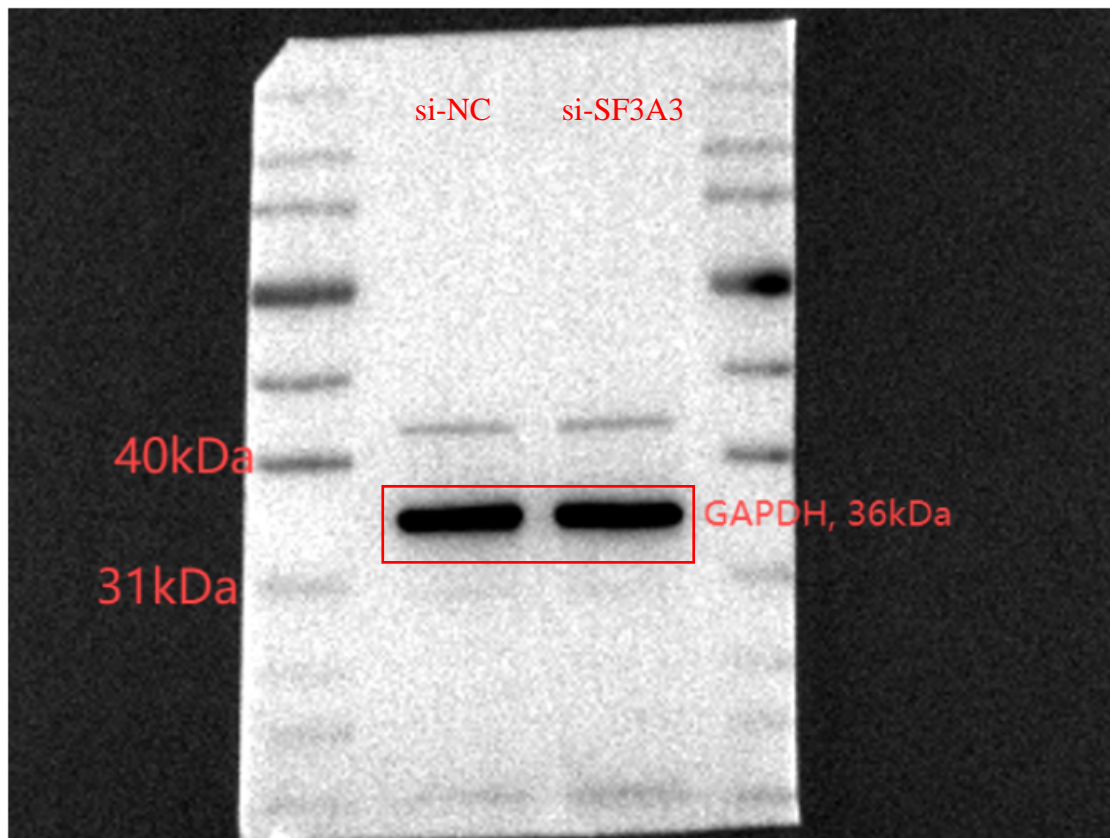

3:

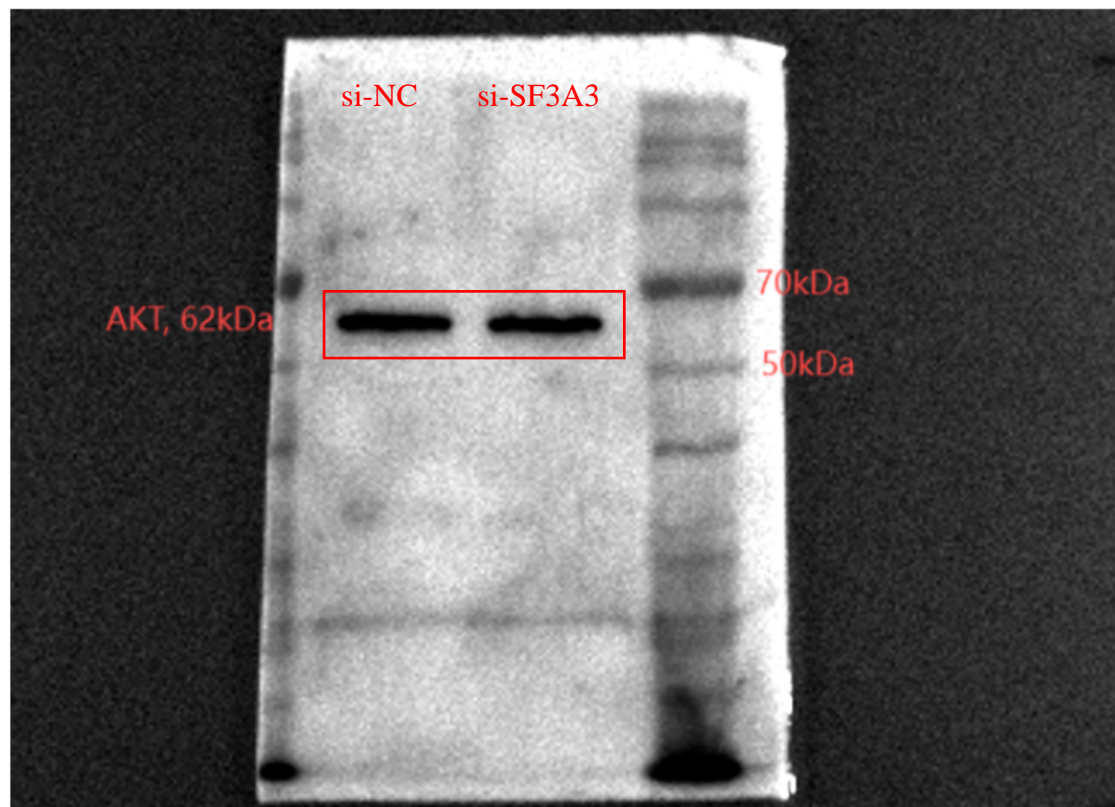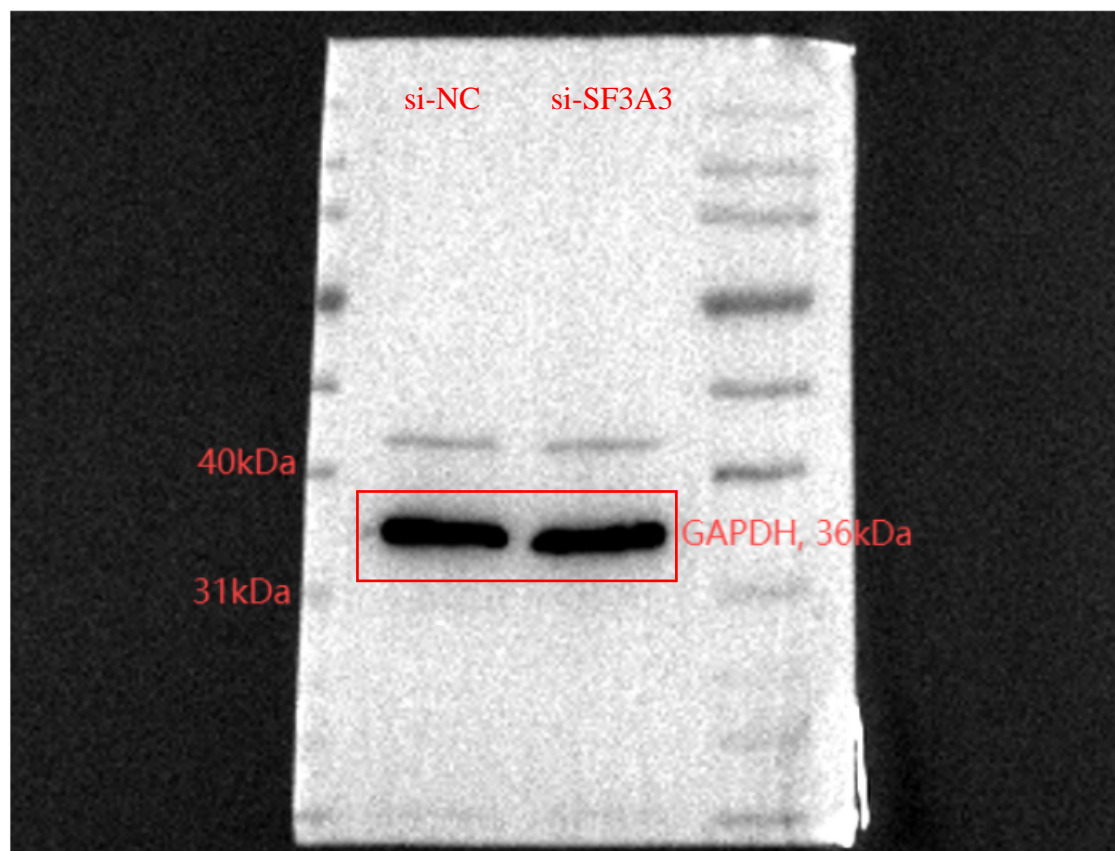

p-AKT:

1:

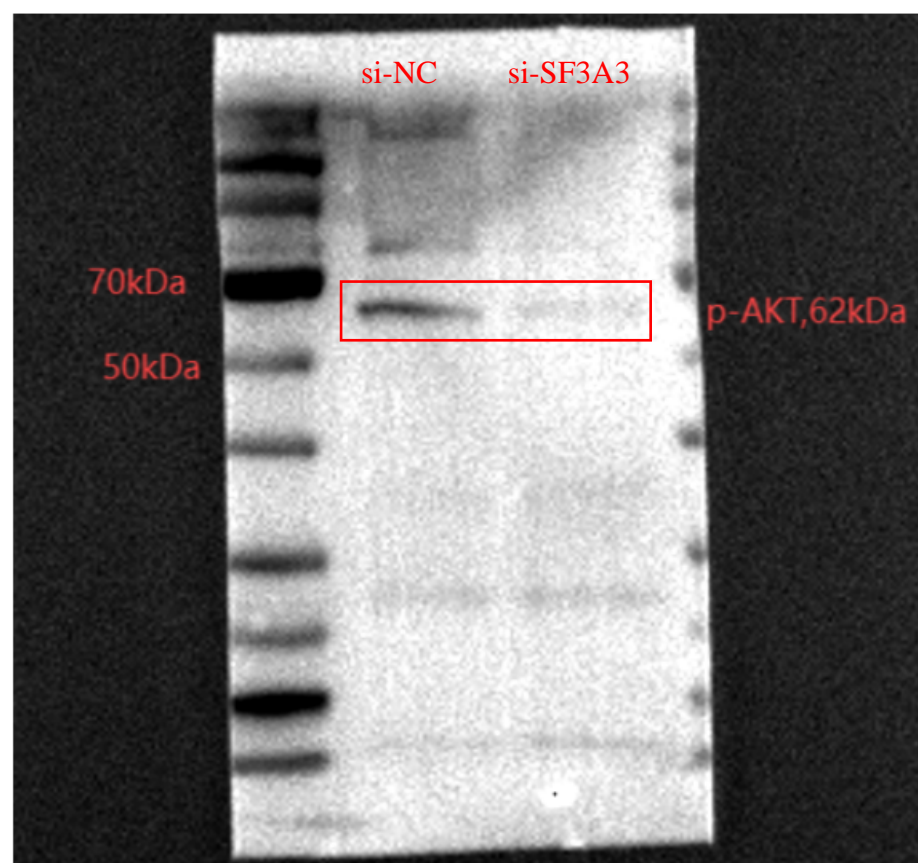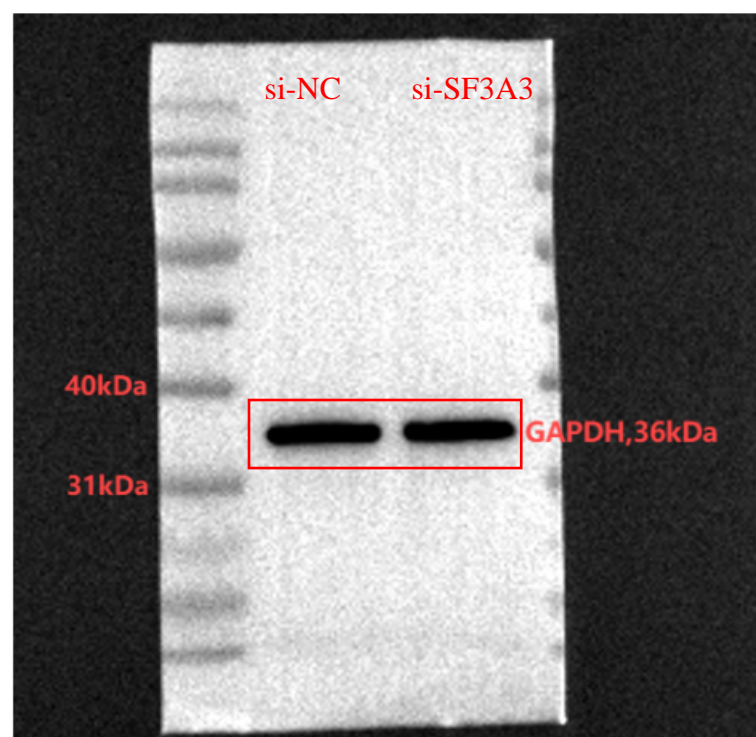

2:

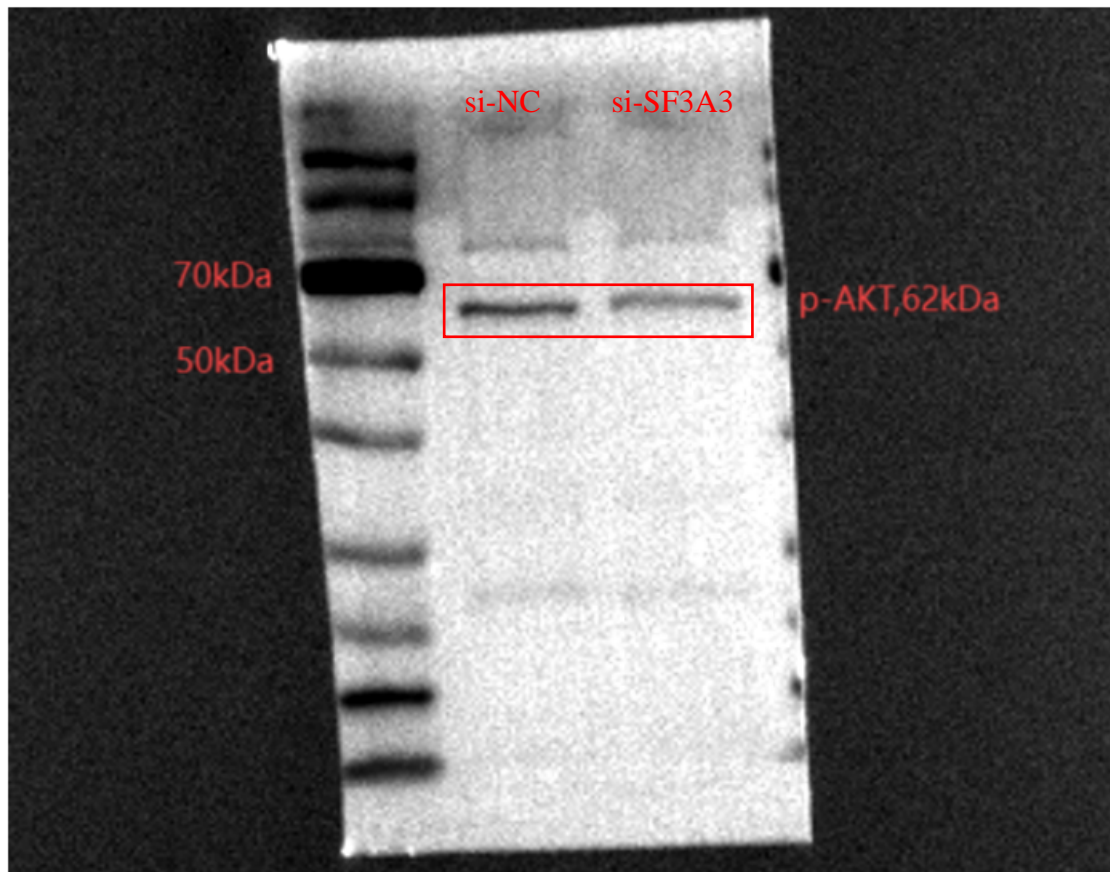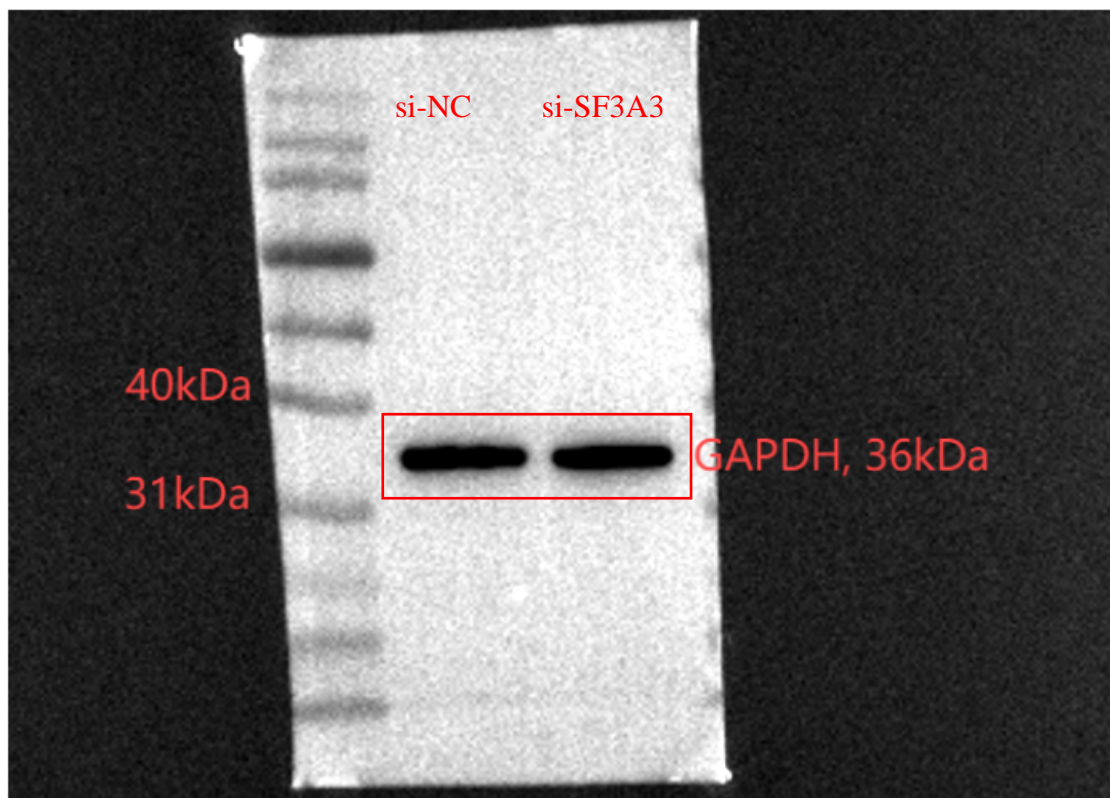

3:

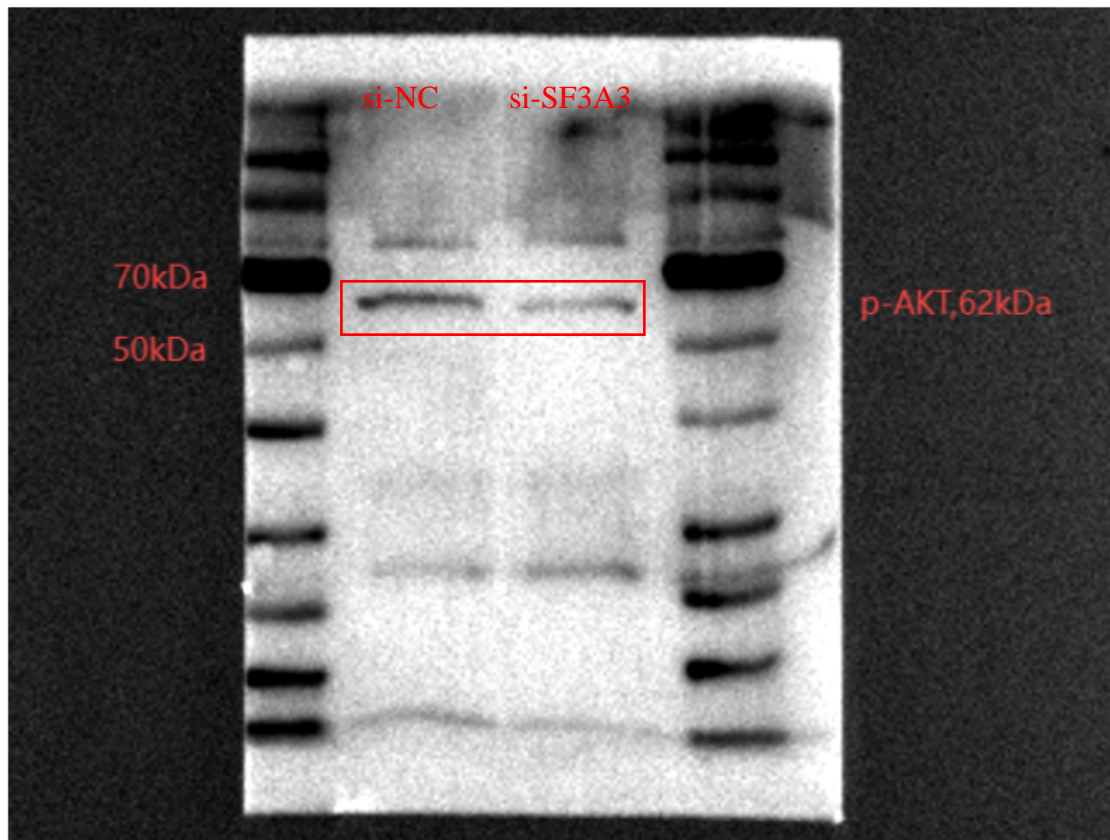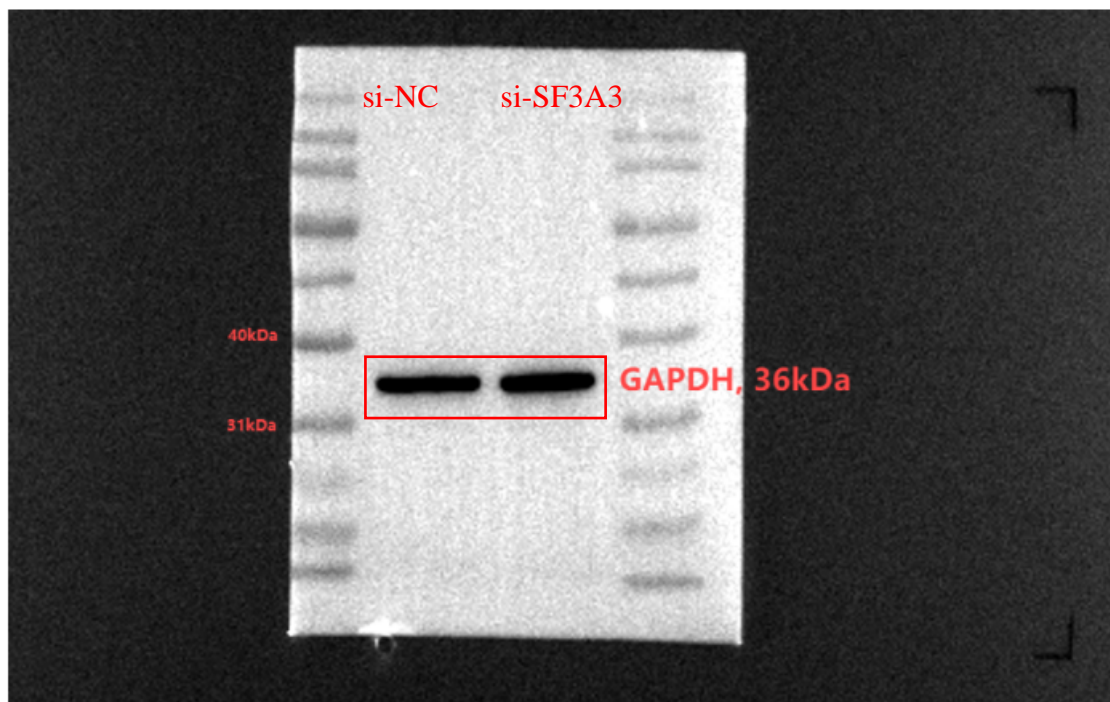

SF3A3:

1:

si-NC

si-SF3A3

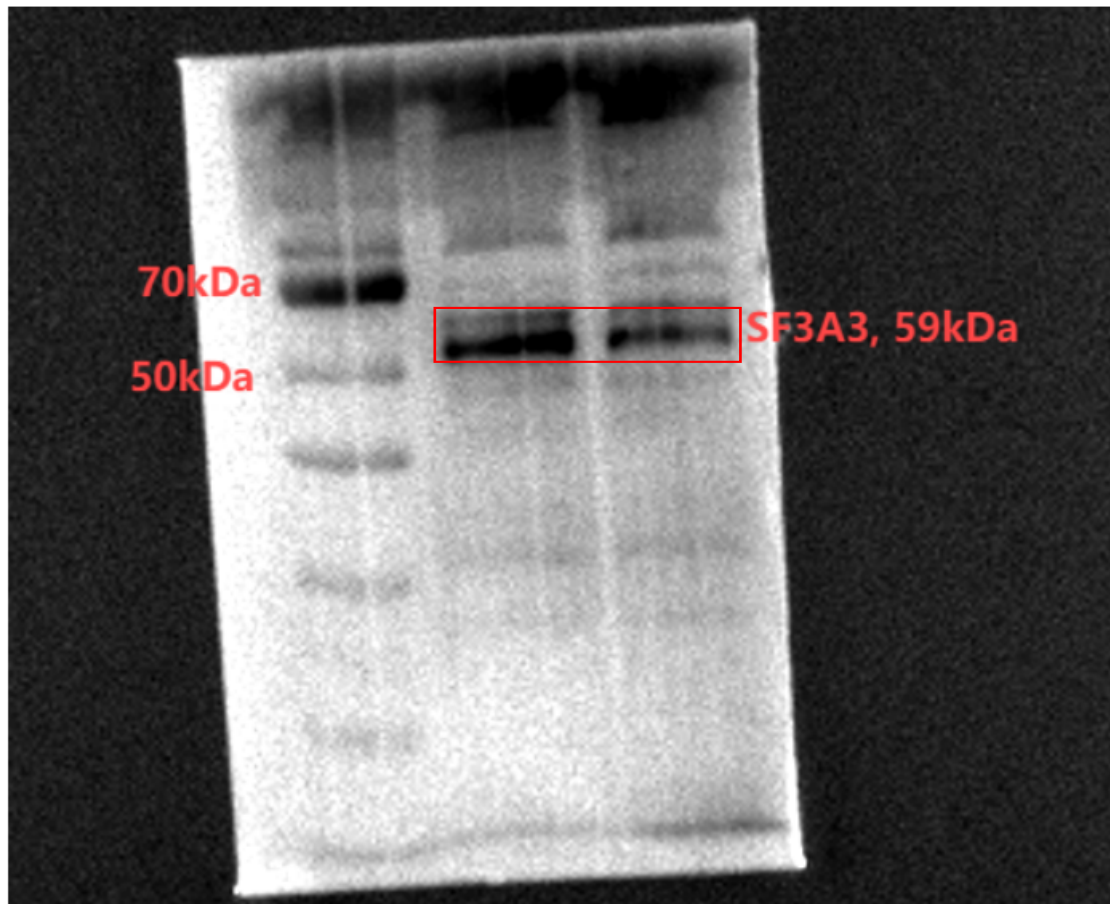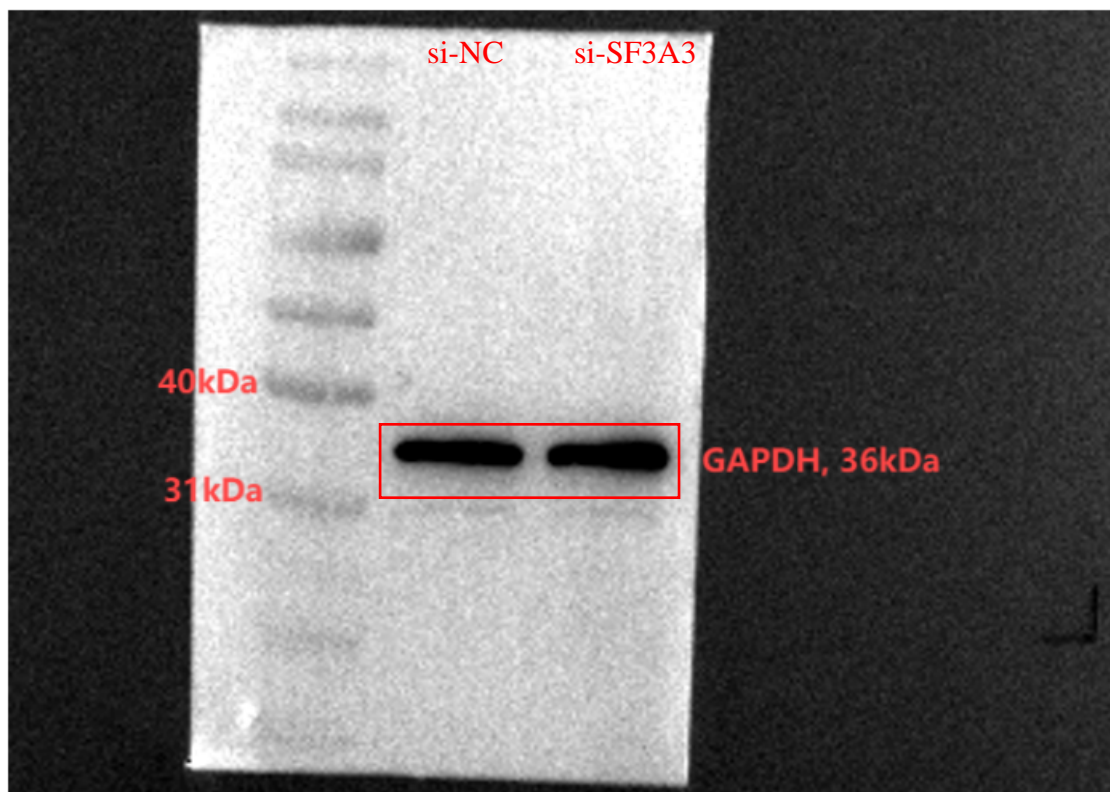

2:

si-NC

si-SF3A3

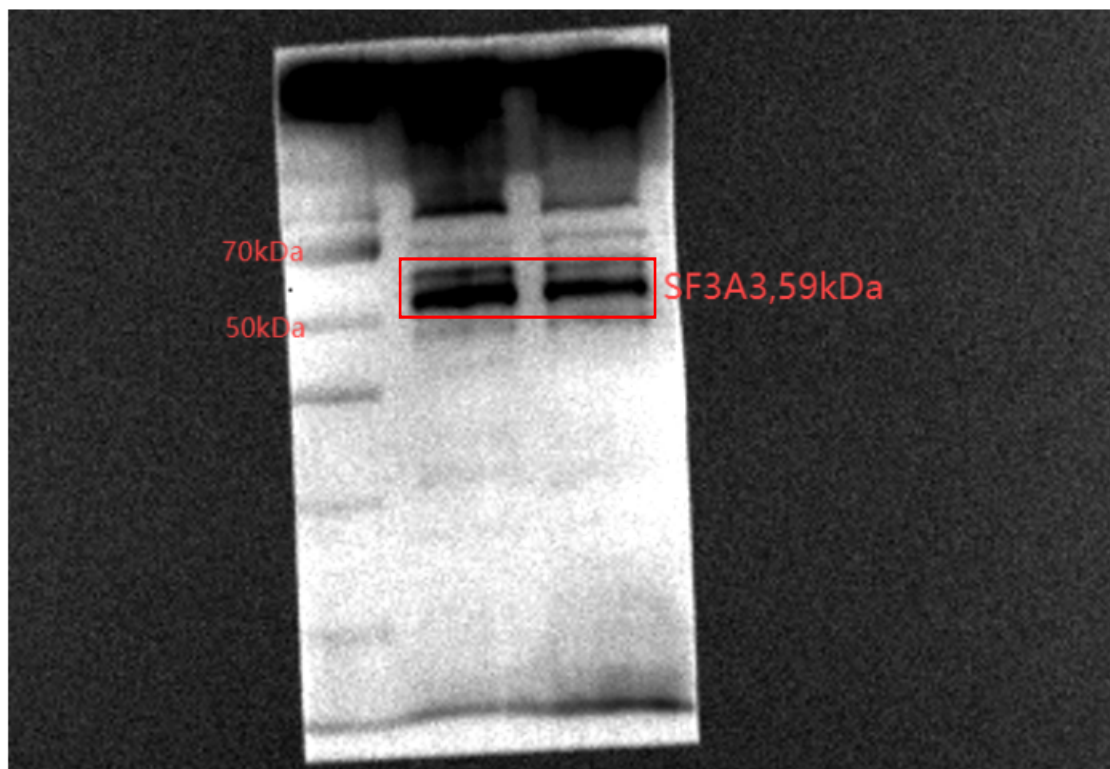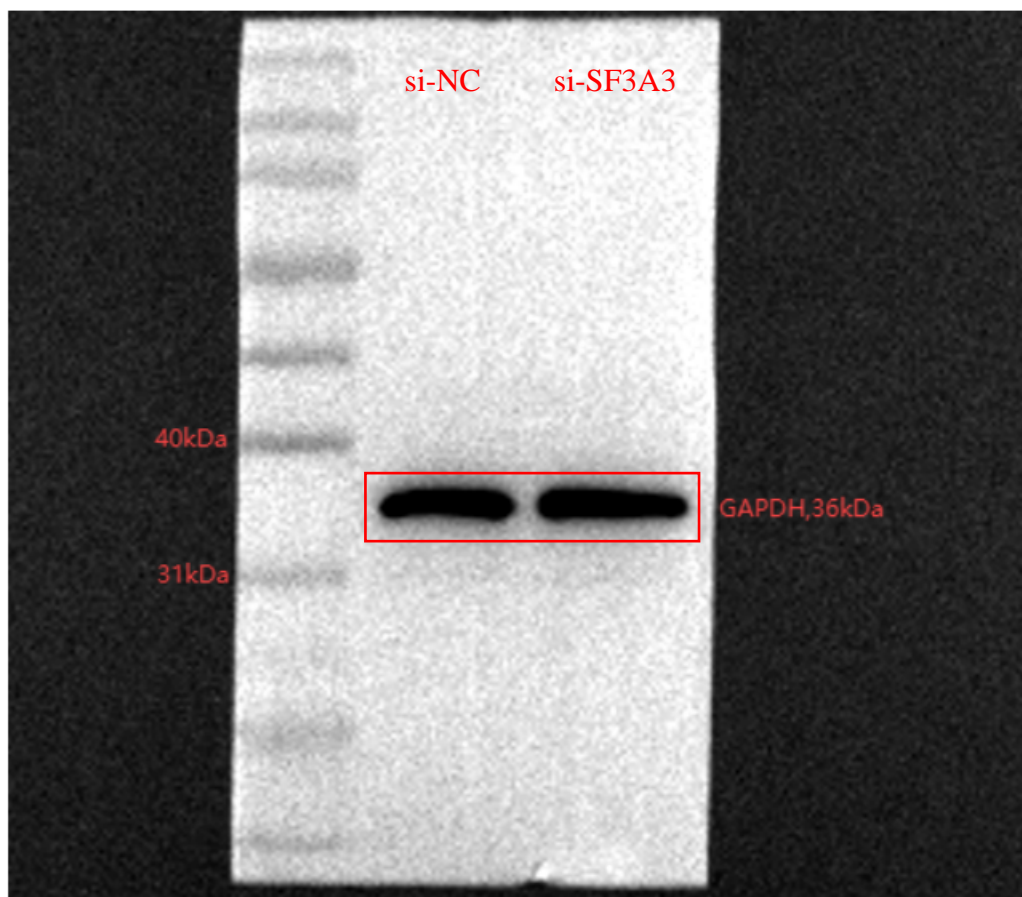

3:

si-NC si-SF3A3

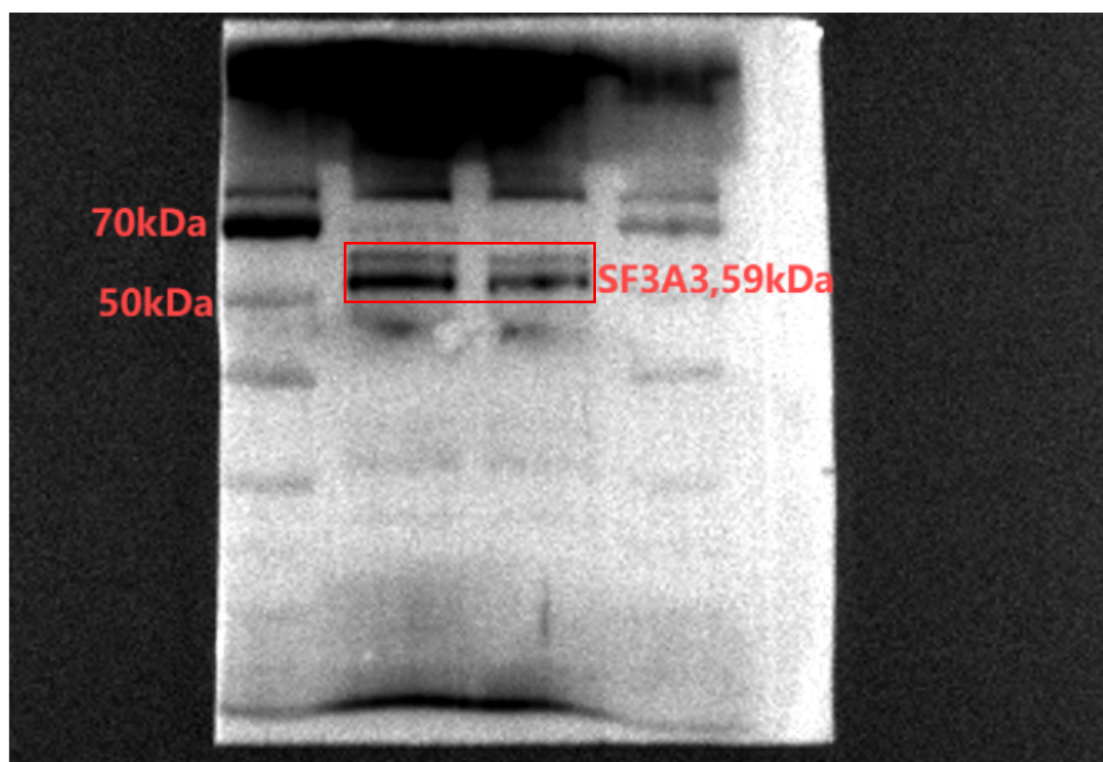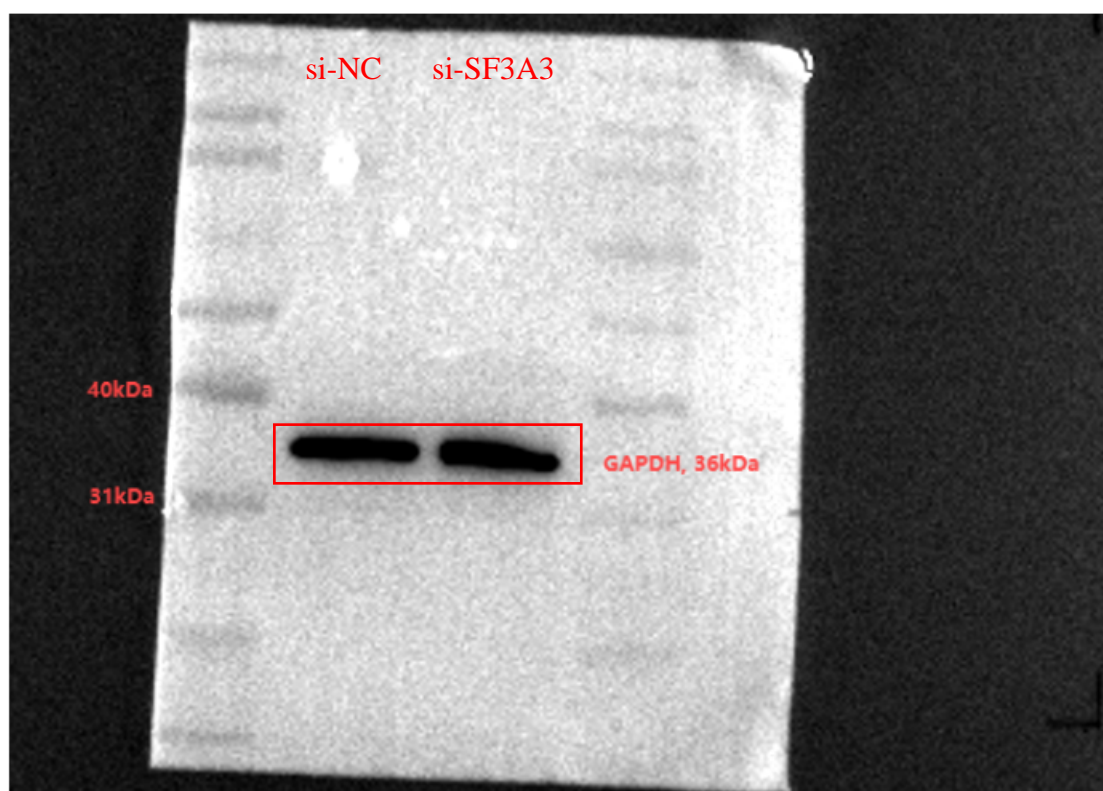

Supplement: S1 File — (PDF) [file pone.0323559.s004.pdf]
